# Fine-Tuning, Retrieval-Augmented Generation, and Hybrid Adaptation of Language Models for Clinical Decision-Making in Health Care: Systematic Review

**DOI:** 10.2196/104092

**Published:** 2026-09-17

**Authors:** Anshum Patel, Yugant Khand, Sai Krishna Vallamchetla, Pengze Li, Cui Tao, Joseph Cheung

**Affiliations:** 1 Division of Pulmonary, Allergy and Sleep Medicine, Mayo Clinic, 4500 San Pablo Road South, Jacksonville, FL, United States, 1 904-953-2000; 2 Department of Neurology, Mayo Clinic in Florida, Jacksonville, FL, United States; 3 Department of Artificial Intelligence and Informatics, Mayo Clinic in Florida, Jacksonville, FL, United States

**Keywords:** AI, language models, large language models, LLMs, small language models, SLMs, generative AI, fine-tuning, retrieval-augmented generation, RAG, reinforcement learning, natural language processing, clinical decision support, health care, AI agents, evidence-based medicine, posttraining methods

## Abstract

**Background:**

Large language models (LLMs) demonstrate strong performance on medical knowledge benchmarks, but their safe and effective use in clinical practice depends on posttraining adaptation rather than raw model capability. Fine-tuning, retrieval-augmented generation (RAG), and hybrid approaches are principal strategies for grounding language models in clinical evidence, yet their comparative effectiveness remains unclear.

**Objective:**

This systematic review aims to synthesize evidence on fine-tuning, RAG, and hybrid posttraining strategies for clinical diagnosis and decision-support tasks and to identify strategy-task alignments and methodological features associated with improved performance.

**Methods:**

We conducted a systematic review in accordance with PRISMA (Preferred Reporting Items for Systematic Reviews and Meta-Analyses) 2020 guidelines. PubMed/MEDLINE, Scopus, and Web of Science were searched from January 2018 through May 2026. Eligible studies evaluated transformer-based language models that underwent posttraining adaptation, retrieval augmentation, or both for clinical decision support, diagnosis, triage, risk stratification, or related health care applications. Studies evaluating nonadapted models, non–language-model AI systems, prompt engineering without performance evaluation, or nonclinical applications were excluded. Data extracted included model architecture, adaptation strategy, clinical domain, validation approach, and performance outcomes. Risk of bias was assessed using PROBAST+AI (Prediction model Risk of Bias Assessment Tool for AI). Studies were grouped according to the primary enhancement strategy (fine-tuning or parameter-efficient fine-tuning, RAG, or hybrid approaches), and findings were synthesized descriptively.

**Results:**

Of 1890 identified records, 35 studies published between 2024 and 2026 met eligibility criteria. Enhancement strategies included RAG (17/35, 48.6%), fine-tuning or parameter-efficient fine-tuning (7/35, 20%), and hybrid approaches (11/35, 31.4%). Studies included diverse specialties from oncology, neurology, radiology, mental health, cardiology, ophthalmology, and surgical care. Fine-tuning demonstrated strong performance for task-specific applications, achieving area under the receiver operating characteristic curve values up to 0.912 for cancer detection and area under curve of 0.892 for major depressive disorder prediction, while matching clinician-level diagnostic performance in several studies. RAG improved guideline adherence and diagnostic accuracy, with increases from 71.1% to 92.1% and from 78.9% to 94.7% in guideline-based decision-support tasks. However, benefits were inconsistent across larger reasoning-capable models. Hybrid systems generally achieved the strongest performance in complex clinical workflows, with external validation accuracies exceeding 90% in stroke triage, dermatology, multimodal imaging, and oncology applications. Risk-of-bias assessment identified substantial methodological limitations, with 25 studies judged as high risk, 9 as unclear risk, and only 1 as low risk overall. Common concerns included inadequate external validation, lack of calibration assessment, nonrepresentative participant selection, and insufficient reporting of analytical methods.

**Conclusions:**

Adaptation strategies should align with task needs, using fine-tuning for narrow classification, RAG for guideline-grounded reasoning, and hybrid approaches for complex multimodal tasks. However, the evidence base remains largely retrospective or benchmark-based. Prospective studies with external validation, calibration, and standardized safety reporting are needed before broader clinical use.

## Introduction

Large language models (LLMs) are reshaping medical AI, but their clinical value depends less on raw benchmark performance than on how effectively they are adapted for real-world care. Medical AI has evolved from narrow systems built for single tasks, such as image classification or risk prediction, toward more general foundation models that can work across language, images, and structured clinical data [1-4]. Generative AI accelerated this shift by making language the main interface: LLMs can follow natural-language instructions, summarize records, answer questions, and draft communication in ways that resemble everyday clinical work more closely than earlier back-end prediction systems [5,6]. This broader capability has made LLMs plausible tools for documentation, patient communication, knowledge synthesis, and diagnostic support, while also sharpening concerns about reliability, bias, transparency, and accountability [4-6].

Early medical language model research understandably focused on proof of capability. In MultiMedQA, Flan-PaLM (Google Research) achieved 67.6% accuracy on MedQA, surpassing the previous state of the art by more than 17 percentage points [7]. Med-PaLM 2 (Google Research) later reached 86.5% on MedQA and, in pairwise evaluation of 1066 consumer medical questions, was preferred over physician answers across 8 of 9 clinically relevant cases [7]. Signals of practical value also emerged in communication and summarization tasks: in a JAMA (Journal of the American Medical Association) Internal Medicine study of 195 patient questions, chatbot responses were preferred in 78.6% of evaluations and approximately 4 to 1 overall over physician responses [8], and in a Nature Medicine reader study, the best-adapted LLM summaries were judged equivalent to medical experts in 45% of cases and superior in 36% of cases [9]. Together, these studies established that LLMs can encode substantial clinical knowledge and perform well on selected language-heavy tasks, but they did so mainly under curated conditions [7-10].

Those gains, however, should not be mistaken for clinical readiness. Examination questions and curated vignettes offer complete information and a constrained answer space, and therefore do not fully test uncertainty, evolving context, workflow integration, or downstream effects on clinician performance [11-13]. When evaluations moved closer to real diagnostic reasoning, results became more mixed. In a complex diagnostic challenge, GPT-4 (OpenAI) included the final diagnosis in its differential in 64% of cases and ranked it first in 39% of cases [14]. In another physician-comparison study, GPT-4 achieved higher median Revised-IDEA (Interpretive Summary, Differential Diagnosis, Explanation of Reasoning, and Alternatives) reasoning scores than attendings and residents, but it also produced incorrect reasoning more often than residents [15]. Most importantly, in a randomized clinical trial including 50 physicians, access to a commercial LLM did not significantly improve diagnostic reasoning compared with conventional resources alone [16]. At the same time, current models remain vulnerable to hallucination, poor instruction following, sensitivity to the quantity and order of information, race-based medical content, and the introduction of false details, while narrow evaluations may miss clinically important equity harms [12,13,17,18].

These limitations have shifted the field from asking whether language models can answer medical questions to how they should be improved for dependable clinical use. Fine-tuning aligns a base model with specialized terminology, documentation styles, and target tasks; in clinical summarization and medical evidence synthesis, adapted models have shown meaningful gains and can narrow the gap between open and proprietary systems [9,19]. Retrieval-augmented generation (RAG) addresses a different problem by grounding outputs in external knowledge at inference time, allowing systems to draw on guidelines, literature, or local protocols without retraining the underlying model [10,20]. This approach is especially attractive in medicine, where knowledge changes quickly and local context matters. In a radiology consultation study, adding RAG to a locally deployable model reduced hallucinations from 8% to 0% and improved mean response rank, while preserving the privacy advantages of local deployment [21]. Increasingly, the most clinically plausible systems are hybrid, combining model adaptation, retrieval, and structured prompting rather than relying on any single method alone [10,20,21].

Despite rapid progress, the evidence base for language model improvement methods remains fragmented by specialty, task, base model, data source, and evaluation design [11,12]. In a recent high-level review, 4609 peer-reviewed clinical language model studies were identified, yet only 1048 used real-world patient data and only 19 were prospective randomized trials [11]. This gap is especially important for posttraining methods because choices about fine-tuning, retrieval, and hybrid design affect not only performance but also updatability, privacy, traceability, and fit within regulated clinical workflows. We therefore conducted this systematic review to synthesize the evidence on fine-tuning, RAG, and hybrid posttraining methods for clinical language models. Specifically, our objectives included (1) for which clinical task types does each adaptation strategy produce the largest performance gains relative to a nonadapted model or other comparator; (2) which design choices, including corpus scope, chunking, embedding model, prompting structure, and parameter scale, moderate those gains; and (3) how robust is the underlying evidence when judged by external validation, safety and fairness reporting, and risk of bias (ROB).

## Methods

### Study Design

This systematic review was conducted in accordance with the PRISMA (Preferred Reporting Items for Systematic Reviews and Meta-Analyses) 2020 guidelines [22] to synthesize evidence regarding posttraining and retrieval-augmented strategies designed to improve the performance, reliability, and clinical applicability of LLMs for clinical decision-making and outcomes. The protocol was submitted to PROSPERO (International Prospective Register of Systematic Reviews) in February 2026, and the registration (CRD420261308522) was finalized in May 2026, with screening, data extraction, and manuscript preparation proceeding from initial submission. A systematic framework was selected because the review addresses which categories of clinical task posttraining adaptation improve language model performance relative to nonadapted models, clinicians, or alternative decision-support systems.

### Search Strategy and Information Sources

We conducted a systematic search in PubMed or MEDLINE, Scopus, and Web of Science from January 2018 to May 2026. The year 2018 was selected as the inception date because it corresponds with the emergence of the transformer era in natural language processing, which enabled advances in generative AI for health care applications. The search strategies were combined MeSH and EMTREE terms and free-text words spanning across three concept domains that include (1) language model architectures and generative AI systems including “large language model,” “transformer,” “GPT,” “LLaMA,” “Mistral,” “Claude,” “Qwen,” “Gemini,” “Gemma” “generative AI”; (2) posttraining and adaptation techniques, including “fine-tuning,” “instruction tuning,” “retrieval-augmented generation,” “RAG,” “Supervised Fine-Tuning,” “SFT,” “Direct Preference Optimization,” “Direct Preference Optimization,” “DPO,” “Low-Rank Adaptation,” “LoRA,” “parameter efficient fine tuning,” “PEFT”; and (3) clinical applications and outcomes, including “clinical decision support,” “diagnostic accuracy,” “differential diagnosis,” “triage,” “risk stratification,” “medical reasoning.” We used Boolean operators, truncation syntax, and proximity operators to index each database. There were no language or publication-type filters other than those specified in the eligibility criteria during the search stage. The complete search strategy for each database is provided in Table S1 in Multimedia Appendix 1.

### Eligibility Criteria

Studies were included if they met all the following criteria: (1) studies were required to involve clinical datasets derived from electronic health records, radiology or pathology reports, laboratory results, discharge summaries, structured symptom descriptions, clinical vignettes, clinical textbooks, or multimodal clinical datasets across any health care domain; and (2) the study was required to evaluate at least one transformer-based language model, including but not limited to GPT, LLaMA (Meta AI), Mistral, Claude (Anthropic), Gemini (Google DeepMind), or related architectures that had undergone a posttraining adaptation or retrieval augmentation strategy. Eligible adaptation approaches included supervised fine-tuning (SFT), instruction tuning, domain adaptation, reinforcement learning or alignment optimization, direct preference optimization, parameter-efficient fine-tuning (PEFT), low-rank adaptation (LoRA), quantized low-rank adaptation (QLoRA), RAG, multimodal retrieval pipelines, or hybrid architectures integrating retrieval and fine-tuning mechanisms. Studies using retrieval augmentation without parameter updating were also eligible when retrieval mechanisms functioned as model-enhancement strategies intended to improve factual grounding, guideline concordance, retrieval fidelity, or hallucination mitigation in medical decision-support tasks such as diagnosis, differential diagnosis generation, clinical reasoning, triage, risk stratification, treatment recommendation support, or prognosis prediction. We also included studies addressing preoperative and postoperative clinical decision-making in surgical or perioperative contexts, including diagnosis, complication detection, risk stratification, and treatment-planning support. Studies were excluded when the model’s target was intraoperative execution, such as instrument guidance, operative-step recognition, or technical performance assessment. We use the term language model throughout for transformer-based generative and encoder architectures, encompassing both LLMs and smaller domain-specific language models. Preprints were eligible if they reported complete methods and quantitative results, and were assessed for ROB on the same basis.

Studies were excluded if they evaluated a baseline language model without any posttraining adaptation; or (1) used traditional machine learning systems or deep-learning systems without a language model component; or (2) used chatbot or theoretical frameworks without implementation and evaluation; or (3) evaluated prompt engineering in isolation without performance data or reported only nonclinical computational benchmarks; or (4) were conducted in surgical or perioperative settings but focused primarily on the technical conduct or intraoperative performance of a surgical procedure rather than on diagnostic or decision-support tasks; or (5) were reviews, editorials, commentaries, conference abstracts, or study protocols without results were excluded.

The studies were required to report at least one evaluative performance outcome from the following categories: diagnostic accuracy (top-1 or top-k), sensitivity and/or specificity, area under the receiver operating characteristic curve (AUROC) or area under the precision-recall curve, differential diagnosis ranking accuracy, triage or risk stratification accuracy, scored clinical reasoning quality, calibration metrics, hallucination or erroneous recommendation rates, time-to-diagnosis, or results from external validation cohorts. The comparators included non-posttrained language models, human clinicians at any level of training, other clinical decision-support systems, alternative AI or machine learning–diagnostic systems, and standard-of-care diagnosis.

### Screening and Selection

All identified records were imported into the Covidence (Veritas Health Innovation Ltd) systematic review software to facilitate collaborative screening and reviewer blinding. Title and abstract screening were performed independently by 2 reviewers (AP and YK) after automated deduplication, using predefined eligibility criteria. Discrepancies in selection were resolved through structured discussion or adjudication by a third reviewer (SKV).

### Data Extraction

The data extraction was performed using a standardized extraction instrument within Covidence. The following data elements were extracted for each included study: publication year, country, study design, clinical setting, health care setting, patient population or benchmark context, model openness (open-source, open-weight, or proprietary), base-model architecture, parameter scale, modality (text-only or multimodal), enhancement strategy, prompting methodology, retrieval infrastructure, external knowledge source, embedding models, vector databases, benchmark type, dataset characteristics, validation design, external testing procedures, explainability mechanisms, hallucination mitigation approaches, alignment and safety strategies, computational infrastructure, and primary performance outcomes. Data were extracted independently by 2 reviewers (AP and YK), with discrepancies adjudicated by consensus. For the included study coauthored by members of the review team, screening, data extraction, and ROB assessment were performed independently by YK, with the coauthoring reviewers excluded from its assessment.

### Quality Assessment and Analysis

Given the nature of the included studies (primarily diagnostic accuracy and model comparison studies), the ROB was assessed using PROBAST+AI (Prediction model Risk of Bias Assessment Tool for AI [23]). PROBAST+AI was selected because the included studies primarily evaluated posttrained models for diagnostic reasoning, clinical decision support, disease classification, triage, and risk stratification tasks. We conducted our ROB assessment across four predefined methodological domains: (1) participants and data sources, (2) predictor handling, (3) outcome definition, and (4) analytical methodology. These domains were rated as having low, high, or unclear ROB according to the PROBAST+AI framework, along with applicability concerns and the reasoning for any identified bias [23]. The ROB is summarized in Table S2 in Multimedia Appendix 1.

### Synthesis Approach

There was substantial clinical, methodological, and statistical heterogeneity across the included studies due to the diversity in model architecture, posttraining strategies, clinical tasks, outcome metrics, and evaluation of cohort characteristics. Therefore, the studies were grouped according to primary posttraining strategy: (1) SFT or PEFT or instruction tuning, (2) RAG, and (3) hybrid or multicomponent strategies. A meta-analysis was inappropriate due to the heterogeneity of these technologies and clinical contexts. Data were, therefore, descriptively summarized and synthesized for study characteristics, enhancement strategies, evaluation methods, and clinical performance outcomes. We elaborated on the performance relative to base LLMs and comparators, clinical domain coverage, evidence of external validation, and safety signals within each subgroup. When studies reported text-similarity metrics alongside clinical-accuracy outcomes, these are reported separately throughout this review and on a consistently labeled scale; text-similarity scores reflect surface-level overlap with a reference text and should not be interpreted as measures of diagnostic or clinical correctness.

## Results

### Study Selection

**Figure 1.**
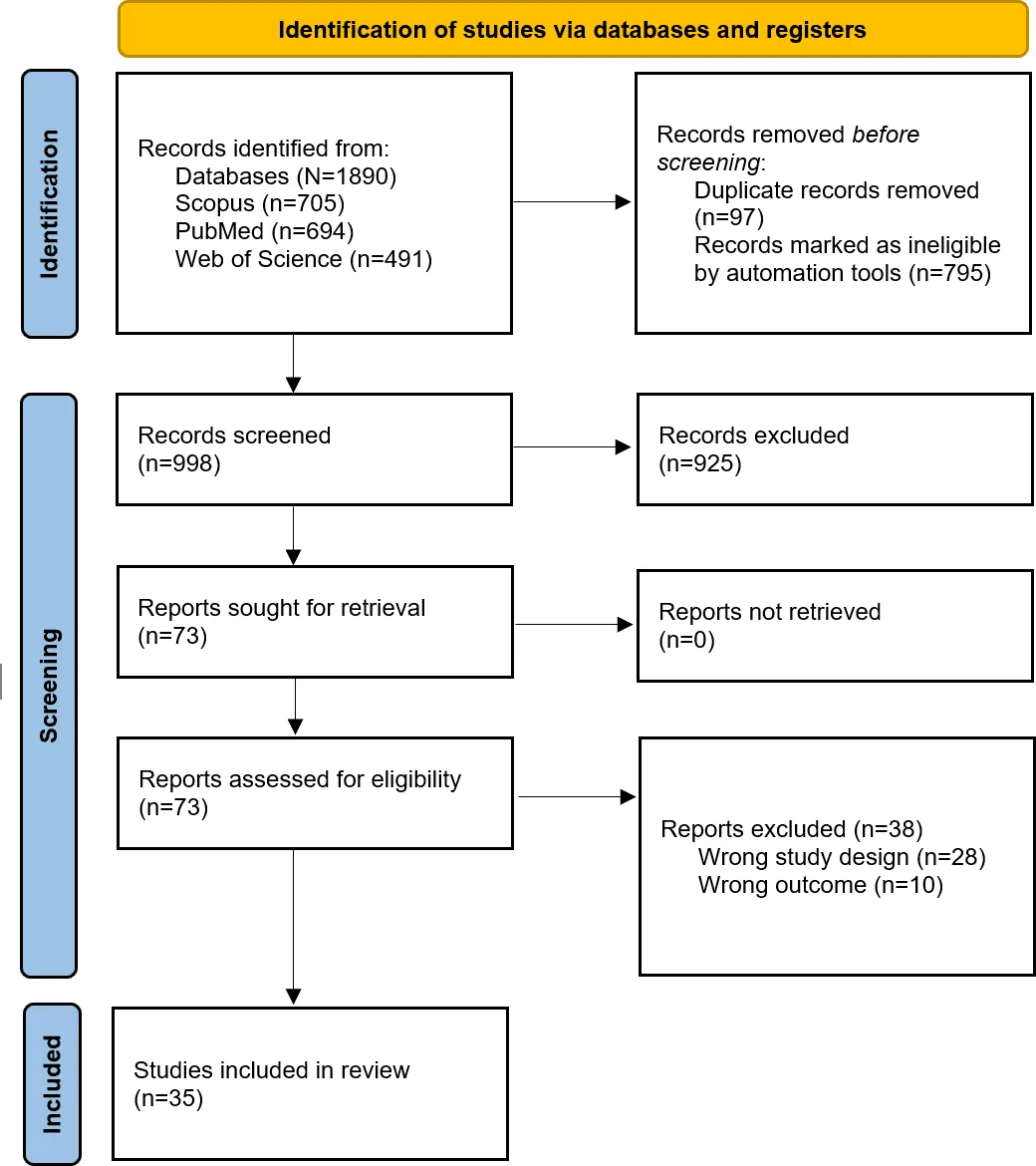
PRISMA (Preferred Reporting Items for Systematic Reviews and Meta-Analyses) flow diagram of study identification, screening, eligibility assessment, and the inclusion process for the systematic review.

The search returned 1890 records. After deduplication and automated filtering, 998 records were screened by title and abstract, of which 925 were excluded as off topic. Full texts were sought for 73 reports; of these, 38 were excluded (28 wrong study design and 10 wrong outcome). The remaining 35 studies published between 2024 and 2026 met inclusion criteria (Figure 1) [24-58]. Key characteristics of included studies are summarized in Table S3 in Multimedia Appendix 1.

### Study Characteristics

**Figure 2.**
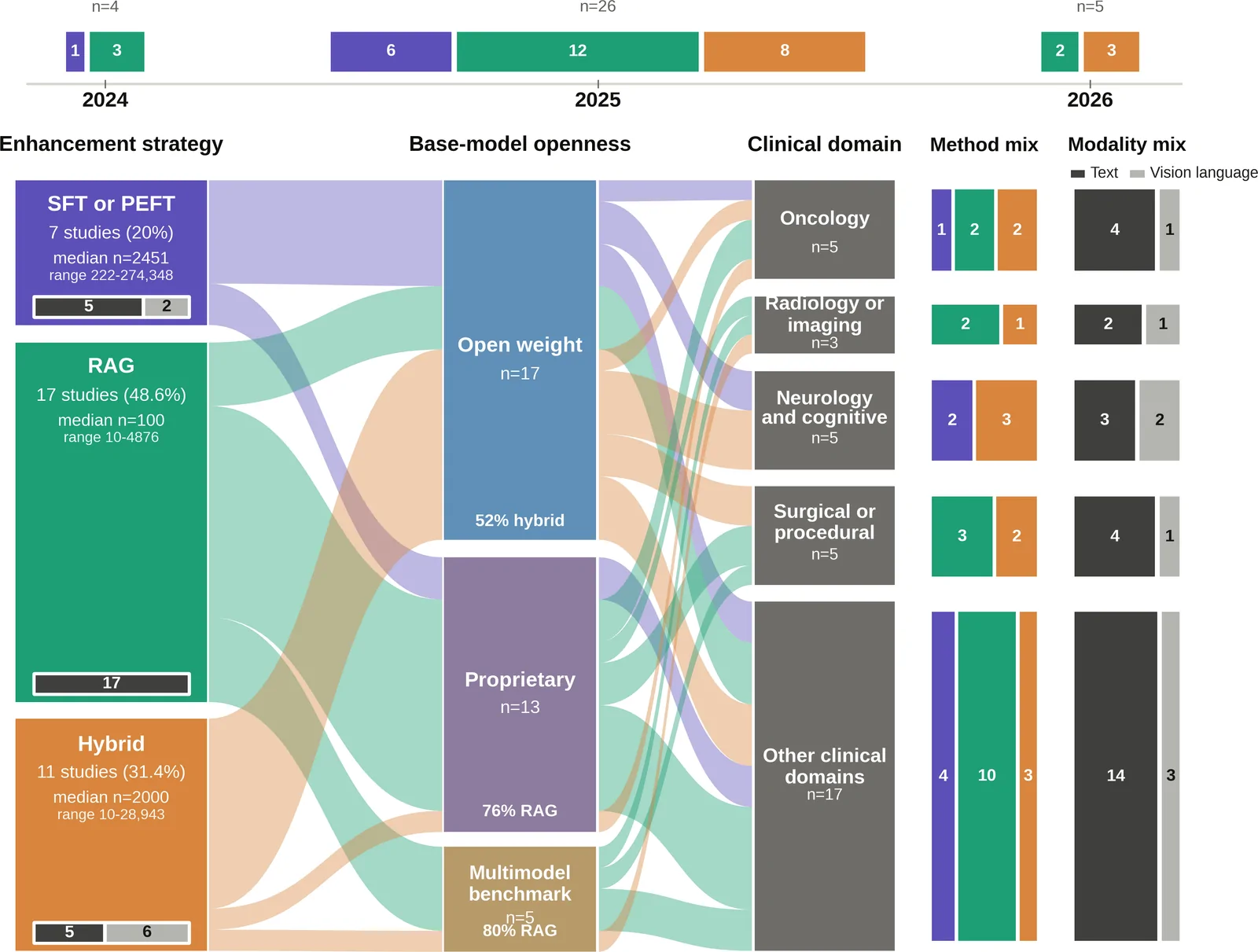
Mapping the landscape of clinical language model adaptation strategies by publication year, enhancement strategy, model openness, clinical domain, and data modality. Ribbon width is proportional to the number of included studies. Strategy nodes report study counts and cohort-size statistics; the right panels summarize method and modality composition within each clinical domain. PEFT: parameter-efficient fine-tuning; RAG: retrieval-augmented generation; SFT: supervised fine-tuning.

Across the included 35 studies, the evaluated enhancement strategies fell into 3 families: SFT or PEFT (n=7, 20%), RAG (n=17, 48.6%), and hybrid pipelines that combined RAG and fine-tuning with structured prompting (n=11, 31.4%; Figure 2) [24-58]. In-silico benchmarks predominated (n=22), followed by retrospective electronic health record–based model-development studies (n=8), prospective proof-of-concept evaluations (n=4), and 1 multicenter retrospective cohort. Clinical domains were heterogeneous, with the most frequent being oncology (n=5), diagnostic radiology (n=3), and neurological and cognitive disorders (n=5); mental health, cardiology, dermatology, ophthalmology, and emergency triage each contributed 2 studies; surgical specialties (perioperative care, hand surgery, microsurgery, and dentistry or oral surgery) contributed 5; and single studies covered pediatrics, rare disease, rehabilitation, urology, sleep medicine, pharmacy, and endocrinology. Studies originated from 12 countries and 1 international federated consortium, with the largest contributions from the United States (n=9), China (n=7), Germany (n=6), and multinational collaborations (n=4); the remainder came from Japan and Singapore (n=2, each), and from Israel, Italy, Canada, Thailand, and Taiwan (n=1, each).

Thirteen studies used proprietary frontier models (GPT-3.5 to GPT-4.5, OpenAI o1 or o3-mini, Claude 3 and 3.5, Gemini 1.5/2.0, Med-PaLM 2, DeepSeek R1, Grok 3 [SpaceXAI]). Seventeen used open-source or open-weight models (LLaMA-2/3 at 7B-70B, Mistral and Mixtral 8×7B, Gemma 2 27B [Google], Qwen 2/2.5/3 [Alibaba Cloud] at 4B-235B, ChatGLM-6B [Zhipu AI] and GLM4-9B [Zhipu AI], Japanese BERT [bidirectional encoder representations from transformers], GatorTron, and InstructBLIP-FLAN-T5-XL [Salesforce AI Research]). Five used mixed or hybrid model stacks. Parameter sizes ranged from approximately 110M to 235B, with 7B to 14B configurations most common. Eight studies were multimodal, integrating text with magnetic resonance imaging, computed tomography, dermoscopy, panoramic radiographs, electrocardiogram (ECG) images, or PubMed figures and tables.

### Adaptation Strategies

Seven studies used fine-tuning as the primary strategy, with LoRA and its variant QLoRA emerging as the de facto standard for parameter-efficient adaptation [24-30]. Fine-tuning was most effective for narrow, well-defined classification tasks where labeled data could be assembled at scale. An instruction-tuned LLaMA-2-7B model classified circulating-tumor-DNA fragmentome features with an external AUROC of 0.912 for cancer detection and 0.938 for hepatocellular carcinoma [24]. A fine-tuned Japanese BERT model flagged newly identified acute infarcts on free-text radiology reports with macrosensitivity of 0.918 and an inference time of 0.115 seconds per patient [26]. At the lower data extreme, a vision fine-tune of GPT-4o trained on only 20 labeled ECGs achieved 79.9% accuracy for detecting reduced left ventricular ejection fraction surpassing average clinician performance, although a dedicated convolutional network remained superior at 89.1% [25]. Fine-tuned GPT-3 generated pediatric differential diagnoses comparable to pediatricians (87.3% vs 91.3%; *P*=.47), using only 350 rural-clinic encounters [29]. At the opposite end of the data spectrum, large-scale instruction tuning of LLaMA 3.1-70B on 274,348 UK Biobank participants outperformed conventional machine learning baselines for major depressive disorder, with an area under curve of 0.892 [30].

RAG was the most common standalone strategy, used in 17 studies, and was applied to 2 dominant use cases: guideline-grounded question answering and literature-based decision support [31-47]. Gains over nonaugmented baselines were largest when the retrieval corpus was authoritative and tightly scoped to the clinical question. Embedding the 2020 European Society of Cardiology acute coronary syndrome guideline raised DeepSeek R1 accuracy from 78.9% to 94.7% and ChatGPT-4o from 71.1% to 92.1% [34]. A trauma-radiology chatbot grounded in a curated reading list improved diagnostic accuracy from 93% to 100% and grading accuracy from 48% to 87% [38]. A guideline-grounded urology pipeline reached 95.5% concordance for prostate-specific–antigen testing recommendations, compared with 62.3% closed-book and 74.1% open-book accuracy among junior clinicians [45]. Specialty-corpus RAG also produced consistent benefits in ophthalmology [32,35], rare disease diagnosis [33], microsurgery and hand surgery [42,43], and radiation oncology [44]. Notably, RAG did not always help. In a 2000-case MIMIC-IV (Medical Information Mart for Intensive Care-IV) evaluation, the strongest standalone Claude 3.5 Sonnet workflow outperformed its RAG-augmented counterpart on overall accuracy [39], and a German emergency-department study found that semantic retrieval introduced formal errors in 23% of responses despite faster retrieval, leaving the nonretrieval Mixtral baseline highest in physician-rated usefulness [40]. Two important moderators of RAG performance were model scale and reasoning capacity. Smaller models were destabilized by retrieval noise unless the corpus was structured and a hybrid sparse-dense index was used; structured table-of-contents–aligned chunks combined with BM25 plus PubMedBERT-dense retrieval reversed a 7.1% accuracy drop in Llama-3-8B and produced a 6.1% mean Top-1 gain on clinical sleep-medicine cases [47]. Reasoning models such as DeepSeek R1 and OpenAI o-series gained little or nothing from retrieval, suggesting that internal chain-of-thought capacity can substitute for some forms of external grounding [44].

### Hybrid and Multicomponent Pipelines

Eleven studies combined fine-tuning, retrieval, and structured prompting into a single pipeline, and these hybrid systems consistently produced the highest clinical-utility scores for complex tasks that required both representation learning and external knowledge grounding. A ChatGLM-6B system that combined LoRA instruction tuning with tool chaining and structured emergency notes correctly classified stroke vs nonstroke in 99.0% of internal cases and 95.5% and 79.1% in 2 external cohorts, with ischemia-vs-hemorrhage accuracy above 97% even on external data [53]. A multimodal Qwen2-VL pipeline for osteonecrosis of the jaw integrated panoramic-radiograph segmentation, LoRA fine-tuning, and visual question answering to reach 96.0% expert-rated accuracy, exceeding junior surgeons (88.4%) and approaching senior surgeons (99.6%) [48]. A federated multimodal dermatology system achieved 90.2% diagnostic accuracy across 11 lesion types and 93.3% benign-vs-malignant accuracy on a 4452-image external Stanford and MIDAS (Multimodal Image Dataset for AI-based Skin Cancer) cohort, while keeping image processing local [57]. A liver-cancer assistant that fused small-model image features, retrieval over the CSCO (Chinese Society of Clinical Oncology) Liver Cancer Guidelines, and a doctor-style 3-step chain-of-thought prompt raised expert-quality scores for image interpretation from 5.9 to 7.2 and treatment-plan reasonableness from 4.2 to 6.5 [54]. Other hybrid systems demonstrated similar synergies in Alzheimer disease [51,52], ophthalmology [50], brain-metastasis magnetic resonance imaging reporting [55], laryngeal-cancer Bayesian network modeling [56], and outpatient diabetes decision support [58].

### Importance of Prompting Strategy

Prompting was treated in many studies as a first-class enhancement lever rather than a secondary detail, and prompt design changes alone produced clinically meaningful shifts in performance. Adding a brief expert-persona preprompt raised guideline-based dental endocarditis-prophylaxis accuracy from 83.6% to 90.0% across 7 frontier models, and progressive least-to-most prompting outperformed simple prompts for chronic low-back–pain treatment recommendations [36,46]. For rare-disease diagnosis, switching from a base prompt to a prompt-with-explanation template increased GPT-3.5 accuracy from 40% to 43% within the same retrieval pipeline [33]. Structured chain-of-thought prompts were used in nearly every hybrid system; in a perioperative-complication pipeline, the combination of a “think” reasoning field, structured JSON outputs, and targeted single-complication prompts more than doubled micro-*F*_1_ in a 4-billion-parameter Qwen model [49]. Multistep, role-based prompting mirroring clinician reasoning was central to Wu-2025-liver-cancer, Tung-2025-PSA-RAG, and Lammert-2024-MTB-CoT, and an adaptive prompt-refinement workflow lifted brain-metastasis detection sensitivity from 0.84 to 0.98 [31,45,54,55]. At the same time, prompting had a clear ceiling: when the underlying model lacked the relevant clinical knowledge or the retrieval corpus was inadequate, prompting changes alone could not close the gap [31,40]. The practical pattern was that small, low-cost changes in persona, chain-of-thought, structured output schemas, and example formatting routinely shifted accuracy by 5 to 15 percentage points and frequently determined whether a downstream evaluation crossed clinically meaningful thresholds.

### Datasets and Knowledge Sources

Dataset quality, structure, and curation mattered as much as raw size. Training corpora ranged from 20 ECG images [25] to 274,348 biobank participants [30], and retrieval corpora ranged from 96 peer-reviewed articles [32] to 30 million PubMed abstracts [39]. Across studies, 3 patterns were consistent. First, well-curated, narrowly-scoped corpora outperformed broader corpora for guideline-bound questions; the Tung-2025 prostate-specific antigen pipeline relied on a 239-page guideline set, and the Alexandrou-2025 acute coronary syndrome pipeline relied on a single guideline document, yet both produced near-ceiling accuracies [34,45]. Second, chunking strategy and embedding model choice were major performance levers. A laryngeal-cancer system raised retrieval accuracy from 0.75 to 0.90 by combining recursive chunking with a fine-tuned general text embeddings–large embedding model [56], and table-of-contents–aligned 512-token segments outperformed raw text segmentation in sleep medicine [47]. Domain-tuned or biomedical embeddings (PubMedBERT-dense [Microsoft Research], bge-small-en [Beijing Academy of Artificial Intelligence], fine-tuned general text embeddings–large) generally outperformed off-the-shelf OpenAI embeddings in retrieval-quality metrics. Third, external validation often revealed substantial performance drops, indicating site-specific adaptation may still be necessary even after fine-tuning or RAG; the Song-2025 stroke model fell from 99.0% internal accuracy to 79.1% in 1 external cohort. Privacy-preserving designs, including federated learning [57], local on-premises deployment of small, fine-tuned models [49,50,58], and institution-owned retrieval indexes [41] were a notable design trend in 2025 to 2026 studies, suggesting growing attention to deployment in regulated clinical environments.

### External Validation, Safety, and ROB

PROBAST+AI assessment showed that 25 of 35 (71.4%) studies were judged to be at high ROB, primarily owing to limited external validation, inadequate calibration assessment, and incomplete methodological reporting. Nine (25.7%) studies had an unclear ROB because key analytical details were insufficiently reported, while only 1 (2.9%) study was rated as low ROB across all domains. Most relied exclusively on internal validation using held-out datasets, retrospective cohorts, benchmark datasets, expert-scored vignettes, or synthetic cases and only a minority of studies performed external validation. These findings highlight substantial methodological limitations in the current evidence base and underscore the need for more rigorous validation and transparent reporting of clinical language model adaptation studies. Full domain-level ratings are presented in Table S2 in Multimedia Appendix 1.

## Discussion

### Principal Findings

In this systematic review of 35 studies, posttraining and retrieval-based adaptation consistently improved the clinical performance of language models across a broad range of tasks. However, the magnitude and reliability of improvement depended heavily on the clinical task, data source, prompting strategy, retrieval design, and system architecture. Several main findings emerged. First, SFT and PEFT were most effective for narrow, well-defined classification or generation tasks supported by labeled clinical data. Second, RAG was most effective for guideline-bound or literature-intensive questions, particularly when the retrieval corpus was accurate, relevant, and well-organized. Third, hybrid systems that combined fine-tuning, retrieval, structured prompting, and, in some cases, multimodal feature extraction appeared most suitable for complex clinical decision-support tasks requiring both learned clinical pattern recognition and access to external medical knowledge. Fourth, prompting was not merely a technical detail but an important performance lever, with structured prompts, expert-role framing, chain-of-thought reasoning, and task decomposition often improving model consistency and clinical usefulness. Fifth, dataset quality, corpus structure, chunking strategy, embedding choice, and external validation were central determinants of performance, often mattering as much as model size. These findings suggest that clinical language model adaptation should not be viewed as a competition between these strategies. Rather, each approach addresses a different limitation of general-purpose models.

Fine-tuning improves task-specific behavior by exposing the model to labeled examples from a defined clinical distribution [59]. This was most evident in studies focused on cancer detection from cfDNA-derived features, acute infarct identification from radiology reports, pediatric differential diagnosis generation, cognitive decline detection, and major depressive disorder classification [24,26,27,29,30]. In these settings, the model was asked to perform a constrained task with a relatively clear reference standard. Even modest datasets were often sufficient to improve performance, especially when the task was narrow and the input format was standardized. This supports the practical value of parameter-efficient methods such as LoRA, which can adapt smaller open-weight models without the cost, privacy burden, or infrastructure needs of full model retraining. Across studies, LoRA generally performed comparably to or slightly better than QLoRA, and several reports suggested that even a few hundred well-curated examples were often sufficient to specialize a foundation model for a single clinical task.

RAG addressed the need to ground model output in external, updated, and domain-specific knowledge. The largest gains were seen when the clinical question mapped closely to a trusted corpus [34,35,38,45,47]. In these cases, RAG improved not only answer accuracy but also the traceability of recommendations. This is clinically important because many errors from general models arise not from language fluency but from outdated, incomplete, or nonspecific medical knowledge. However, RAG was not uniformly beneficial. Some studies showed that poorly matched retrieval, noisy chunks, or overly broad corpora could distract the model and worsen performance [39,40,47]. Thus, retrieval should be treated as a clinical engineering problem rather than a simple add-on. Corpus selection, chunking strategy, embedding model choice, reranking, and postretrieval filtering may determine whether RAG improves or degrades clinical output. Performance was also influenced by model scale and reasoning capacity, with smaller models being more sensitive to retrieval noise unless structured chunking and hybrid sparse–dense indexing were used. In contrast, models with strong reasoning showed limited additional benefit from retrieval, suggesting that strong internal reasoning may partially substitute for external grounding in some settings.

Hybrid systems appeared most promising for high-complexity tasks. Real-world decision support rarely depends on a single capability. It requires recognition of patient-specific patterns, knowledge of guidelines, interpretation of multimodal data, and generation of usable recommendations. Hybrid systems may therefore represent the most realistic architecture for deployment, particularly when the goal extends beyond question answering to workflow-level clinical decision support. Across studies, no single component consistently dominated; instead, performance depended on how well fine-tuning, retrieval, prompting, and multimodal inputs were jointly aligned with the clinical task. Three cross-cutting practical principles emerged among different methods. PEFT, particularly LoRA, was the dominant fine-tuning approach and enabled privacy-preserving on-premises deployment of small open-weight models. Embedding choice, chunking, and prompt engineering were comparatively low-cost levers that frequently mattered more than choosing a larger base model. Reasoning-class models reduced but did not eliminate the marginal benefit of retrieval, suggesting that the optimal architecture is increasingly dependent on the specific clinical task rather than on any single dominant adaptation strategy.

A second important observation is that smaller, locally deployable models can perform well when adaptation is carefully designed. Several studies showed that compact open-weight models could approach or exceed larger proprietary systems after targeted fine-tuning, task decomposition, or domain-specific retrieval [49,50,58]. This has practical implications for health care systems, where data privacy, latency, cost, auditability, and local governance are central barriers to implementation. Open-weight models adapted within institutional environments may offer a more feasible pathway for many clinical applications than reliance on externally hosted general-purpose models. The emergence of federated and privacy-preserving approaches further suggests that multi-institutional model development may be possible without centralizing sensitive patient data [57].

Prompting also emerged as a meaningful determinant of performance. Persona prompts, structured chain-of-thought-style reasoning, JSON output schemas, least-to-most prompting, and task decomposition produced measurable gains across several studies [36,46,49,55]. These findings should not be interpreted to mean that prompting alone is sufficient for clinical reliability. Instead, prompt design appears to function as an interface between the clinical task and the model. It can improve consistency, enforce structure, and reduce ambiguity, but it cannot compensate for poor retrieval, weak reference standards, or lack of domain knowledge. For clinical deployment, prompts should be version-controlled, tested across cases, and reported with enough detail to allow reproducibility.

Despite encouraging results, the current evidence base remains early. Most included studies were retrospective, in-silico, or proof-of-concept evaluations. Only a small number used prospective designs, and even fewer assessed real-world workflow integration, clinician behavior, patient outcomes, or downstream safety. Many studies reported accuracy, *F*_1_-score, AUROC, or expert-rated quality, but fewer evaluated calibration, uncertainty, subgroup performance, hallucination rates, or harmful recommendations. External validation was inconsistent, and, when performed, performance sometimes dropped substantially across sites or cohorts. These findings underscore that benchmark performance alone is insufficient for clinical readiness. For decision support systems, future studies should assess not only whether the model is correct but also when it is wrong, whether it knows when to abstain, how errors affect clinicians, and whether use of the system improves patient-relevant outcomes.

### Limitations

This review has limitations. First, meta-analysis was not feasible because of substantial heterogeneity in clinical domains, model architectures, adaptation methods, outcome metrics, and evaluation designs. Our study reports the direction and consistency of reported effects rather than pooled estimates, and the descriptive characterization of strategies, clinical domains, and model families shares features with evidence mapping. Second, most studies were model-development or benchmark studies rather than prospective clinical trials, limiting inference about real-world effectiveness. Third, many studies used simulated vignettes, synthetic cases, or curated datasets, which may overestimate performance compared with routine clinical environments. Fourth, reporting of calibration, fairness, demographic subgroup performance, and safety outcomes was inconsistent. Fifth, the rapid pace of LLM development means that model versions, retrieval tools, and fine-tuning methods may change quickly, limiting the durability of model-specific conclusions. Sixth, although the search combined generic architecture terms with the named-model terms, it was necessarily incomplete, as relevant studies are not consistently described as LLMs in titles or abstracts. We did not assess reporting bias formally, as no established method applies to this class of study, and these findings should be read as indicating the direction rather than the magnitude of benefit. However, the broader task-to-method patterns identified in this review are likely to remain relevant as clinical LLM systems continue to evolve.

### Conclusions

In clinical practice, the performance of LLMs depends less on any single technical approach and more on how these tools are combined to support real clinical decision-making. Fine-tuning helps models adapt to specific clinical tasks, retrieval grounds outputs in current medical knowledge, structured prompting improves clarity and consistency of responses, and multimodal inputs allow incorporation of imaging and other patient data. When used together appropriately, these approaches can improve the usefulness of AI systems in supporting clinicians, but their value ultimately depends on how well they fit into clinical workflows and decision needs. Future research should move beyond isolated benchmark improvements toward prospective, externally validated, and clinically embedded evaluations that prioritize safety, reliability, and patient-centered outcomes.

## Supplementary material

10.2196/104092 Multimedia Appendix 1 Search strategies, risk of bias, and characteristics of included studies.

10.2196/104092 Checklist 1 PRISMA checklist.

## Abbreviations

AUROC: area under the receiver operating characteristic curve
BERT: bidirectional encoder representations from transformers
CSCO: Chinese Society of Clinical Oncology
ECG: electrocardiogram
IDEA: Interpretive Summary, Differential Diagnosis, Explanation of Reasoning, and Alternatives
LLM: large language model
LoRA: low-rank adaptation
MIDAS: Multimodal Image Dataset for AI-based Skin Cancer
MIMIC-IV: Medical Information Mart for Intensive Care-IV
PEFT: parameter-efficient fine-tuning
PRISMA: Preferred Reporting Items for Systematic Reviews and Meta-Analyses
PROBAST+AI: Prediction model Risk of Bias Assessment Tool for AI
PROSPERO: International Prospective Register of Systematic Reviews
QLoRA: quantized low-rank adaptation
RAG: retrieval-augmented generation
ROB: risk of bias
SFT: supervised fine-tuning
