# Supplementary material for: Fine-Tuning, Retrieval-Augmented Generation, and Hybrid Adaptation of Language Models for Clinical Decision-Making in Health Care: Systematic Review
Source: J Med Internet Res. 2026 Sep 17;28:e104092. doi: 10.2196/104092 (PMC13585368; doi:10.2196/104092)
Supplement: Supplementary file 2 [file jmir-v28-e104092-s001.docx]

**SUPPLEMENTARY FILE**

**Table S1. Database Search Strategies**

| **Database** | **Strategy** |
| --- | --- |
| **1. PubMed/MEDLINE** | ("Artificial Intelligence"[Mesh] OR "Natural Language Processing"[Mesh] OR "Machine Learning"[Mesh] OR "artificial intelligence"[tiab] OR "generative artificial intelligence"[tiab] OR "generative AI"[tiab] OR "large language model*"[tiab] OR "large-language model*"[tiab] OR LLM[tiab] OR LLMs[tiab] OR "language model*"[tiab] OR "foundation model*"[tiab] OR "generative language model*"[tiab] OR "transformer model*"[tiab] OR "transformer-based"[tiab] OR transformer*[tiab] OR GPT[tiab] OR GPT-2[tiab] OR GPT-3[tiab] OR GPT-4[tiab] OR GPT-5[tiab] OR ChatGPT[tiab] OR LLaMA[tiab] OR Llama[tiab] OR Mistral[tiab] OR Claude[tiab] OR Qwen[tiab] OR Gemini[tiab] OR Gemma[tiab] OR PaLM[tiab] OR BERT[tiab] OR BioBERT[tiab] OR ClinicalBERT[tiab] OR PubMedBERT[tiab] OR BioGPT[tiab] OR Med-PaLM[tiab] OR MedPaLM[tiab] OR "domain-specific language model*"[tiab] OR "clinical language model*"[tiab] OR "medical language model*"[tiab] OR "healthcare language model*"[tiab])  AND  ("fine-tuning"[tiab] OR fine-tun*[tiab] OR "fine tuning"[tiab] OR "supervised fine-tuning"[tiab] OR SFT[tiab] OR "instruction tuning"[tiab] OR "instruction-tun*"[tiab] OR "instruction fine-tuning"[tiab] OR "domain adaptation"[tiab] OR "domain-adapted"[tiab] OR "model adaptation"[tiab] OR "post-training"[tiab] OR "post training"[tiab] OR "post-trained"[tiab] OR "transfer learning"[tiab] OR "reinforcement learning"[tiab] OR "reinforcement learning from human feedback"[tiab] OR RLHF[tiab] OR "reinforcement learning from AI feedback"[tiab] OR RLAIF[tiab] OR "direct preference optimization"[tiab] OR DPO[tiab] OR "preference optimization"[tiab] OR "alignment optimization"[tiab] OR "parameter-efficient fine-tuning"[tiab] OR "parameter efficient fine tuning"[tiab] OR PEFT[tiab] OR "parameter-efficient adaptation"[tiab] OR "low-rank adaptation"[tiab] OR LoRA[tiab] OR QLoRA[tiab] OR "quantized low-rank adaptation"[tiab] OR "retrieval-augmented generation"[tiab] OR "retrieval augmented generation"[tiab] OR RAG[tiab] OR "retrieval-augmented"[tiab] OR "retrieval augmented"[tiab] OR "retrieval-enhanced"[tiab] OR "retrieval enhanced"[tiab] OR "retrieval-based augmentation"[tiab] OR "retrieval-based"[tiab] OR "knowledge augmentation"[tiab] OR "knowledge-enhanced"[tiab] OR "external knowledge"[tiab] OR "external knowledge retrieval"[tiab] OR "knowledge retrieval"[tiab] OR "multimodal retrieval"[tiab] OR "retrieval pipeline*"[tiab] OR "hybrid architecture*"[tiab])  AND  ("clinical"[tiab] OR "medical"[tiab] OR "healthcare"[tiab] OR "health care"[tiab] OR "patient care"[tiab] OR "clinical practice"[tiab] OR "clinical setting*"[tiab] OR "medical decision*"[tiab] OR "clinical decision*"[tiab] OR "decision support"[tiab] OR "clinical decision support"[tiab] OR "clinical decision-support"[tiab] OR "diagnos*"[tiab] OR "differential diagnos*"[tiab] OR "triage"[tiab] OR "risk stratification"[tiab] OR "risk prediction"[tiab] OR "prognos*"[tiab] OR "treatment recommendation*"[tiab] OR "therapeutic recommendation*"[tiab] OR "management recommendation*"[tiab] OR "clinical reasoning"[tiab] OR "medical reasoning"[tiab] OR "clinical judgement"[tiab] OR "clinical judgment"[tiab] OR "medical decision making"[tiab] OR "clinical decision making"[tiab] OR "diagnostic reasoning"[tiab] OR "diagnostic support"[tiab] OR "treatment planning"[tiab] OR "clinical prediction"[tiab] OR "patient management"[tiab] OR "perioperative"[tiab] OR "preoperative"[tiab] OR "pre-operative"[tiab] OR "postoperative"[tiab] OR "post-operative"[tiab] OR surgical[tiab] OR surgery[tiab] OR "clinical vignette*"[tiab] OR "electronic health record*"[tiab] OR EHR[tiab] OR EHRs[tiab] OR "electronic medical record*"[tiab] OR EMR[tiab] OR "radiology report*"[tiab] OR "pathology report*"[tiab] OR "laboratory result*"[tiab] OR "discharge summar*"[tiab])  AND  ("2018/01/01"[Date - Publication] **:** "2026/05/31"[Date - Publication]) |
| **2. Scopus** | ("large language model*" OR "large-language model*" OR "language model*" OR LLM OR LLMs OR "foundation model*" OR "generative language model*" OR "generative AI" OR "generative artificial intelligence" OR "artificial intelligence" OR transformer* OR "transformer model*" OR "transformer-based" OR GPT OR "GPT-2" OR "GPT-3" OR "GPT-4" OR "GPT-5" OR ChatGPT OR LLaMA OR Llama OR Mistral OR Claude OR Qwen OR Gemini OR Gemma OR PaLM OR BERT OR BioBERT OR ClinicalBERT OR PubMedBERT OR BioGPT OR "Med-PaLM" OR MedPaLM OR "clinical language model*" OR "medical language model*" OR "healthcare language model*" OR "domain-specific language model*")  AND  ("fine tuning" OR "fine-tuning" OR "fine tun*" OR "supervised fine-tuning" OR SFT OR "instruction tuning" OR "instruction-tuning" OR "instruction fine-tuning" OR "domain adaptation" OR "domain-adapted" OR "model adaptation" OR "post-training" OR "post training" OR "post-trained" OR "transfer learning" OR "reinforcement learning" OR RLHF OR "reinforcement learning from human feedback" OR RLAIF OR "reinforcement learning from AI feedback" OR "direct preference optimization" OR DPO OR "preference optimization" OR "alignment optimization" OR "parameter-efficient fine-tuning" OR "parameter efficient fine tuning" OR PEFT OR "parameter-efficient adaptation" OR "low-rank adaptation" OR LoRA OR QLoRA OR "quantized low-rank adaptation" OR "retrieval-augmented generation" OR "retrieval augmented generation" OR RAG OR "retrieval-augmented" OR "retrieval augmented" OR "retrieval-enhanced" OR "retrieval enhanced" OR "retrieval-based augmentation" OR "knowledge augmentation" OR "knowledge-enhanced" OR "external knowledge retrieval" OR "knowledge retrieval" OR "multimodal retrieval" OR "retrieval pipeline*" OR "hybrid architecture*")  AND  ("clinical decision support" OR "clinical decision-support" OR "decision support" OR "clinical decision making" OR "clinical decision-making" OR "medical decision making" OR "medical decision-making" OR "clinical reasoning" OR "medical reasoning" OR "diagnostic reasoning" OR "diagnostic support" OR "differential diagnosis" OR "diagnostic accuracy" OR diagnos* OR triage OR "risk stratification" OR "risk prediction" OR prognos* OR "treatment recommendation*" OR "therapeutic recommendation*" OR "management recommendation*" OR "treatment planning" OR "clinical prediction" OR "patient management" OR "clinical judgement" OR "clinical judgment" OR "medical judgement" OR "medical judgment" OR perioperative OR preoperative OR "pre-operative" OR postoperative OR "post-operative" OR surgical OR surgery OR "electronic health record*" OR EHR OR EHRs OR "electronic medical record*" OR EMR OR "radiology report*" OR "pathology report*" OR "laboratory result*" OR "discharge summar*" OR "clinical vignette*") |
| **3. Web of Science** | ("large language model*" OR "large-language model*" OR "language model*" OR LLM OR LLMs OR "foundation model*" OR "generative language model*" OR "generative AI" OR "generative artificial intelligence" OR "artificial intelligence" OR transformer* OR "transformer model*" OR "transformer-based" OR GPT OR "GPT-2" OR "GPT-3" OR "GPT-4" OR "GPT-5" OR ChatGPT OR LLaMA OR Llama OR Mistral OR Claude OR Qwen OR Gemini OR Gemma OR PaLM OR BERT OR BioBERT OR ClinicalBERT OR PubMedBERT OR BioGPT OR "Med-PaLM" OR MedPaLM OR "clinical language model*" OR "medical language model*" OR "healthcare language model*" OR "domain-specific language model*")  AND  ("fine tuning" OR "fine-tuning" OR "fine tun*" OR "supervised fine-tuning" OR SFT OR "instruction tuning" OR "instruction-tuning" OR "instruction fine-tuning" OR "domain adaptation" OR "domain-adapted" OR "model adaptation" OR "post-training" OR "post training" OR "post-trained" OR "transfer learning" OR "reinforcement learning" OR RLHF OR "reinforcement learning from human feedback" OR RLAIF OR "reinforcement learning from AI feedback" OR "direct preference optimization" OR DPO OR "preference optimization" OR "alignment optimization" OR "parameter-efficient fine-tuning" OR "parameter efficient fine tuning" OR PEFT OR "parameter-efficient adaptation" OR "low-rank adaptation" OR LoRA OR QLoRA OR "quantized low-rank adaptation" OR "retrieval-augmented generation" OR "retrieval augmented generation" OR RAG OR "retrieval-augmented" OR "retrieval augmented" OR "retrieval-enhanced" OR "retrieval enhanced" OR "retrieval-based augmentation" OR "knowledge augmentation" OR "knowledge-enhanced" OR "external knowledge retrieval" OR "knowledge retrieval" OR "multimodal retrieval" OR "retrieval pipeline*" OR "hybrid architecture*")  AND  ("clinical decision support" OR "clinical decision-support" OR "decision support" OR "clinical decision making" OR "clinical decision-making" OR "medical decision making" OR "medical decision-making" OR "clinical reasoning" OR "medical reasoning" OR "diagnostic reasoning" OR "diagnostic support" OR "differential diagnosis" OR "diagnostic accuracy" OR diagnos* OR triage OR "risk stratification" OR "risk prediction" OR prognos* OR "treatment recommendation*" OR "therapeutic recommendation*" OR "management recommendation*" OR "treatment planning" OR "clinical prediction" OR "patient management" OR "clinical judgement" OR "clinical judgment" OR "medical judgement" OR "medical judgment" OR perioperative OR preoperative OR "pre-operative" OR postoperative OR "post-operative" OR surgical OR surgery OR "electronic health record*" OR EHR OR EHRs OR "electronic medical record*" OR EMR OR "radiology report*" OR "pathology report*" OR "laboratory result*" OR "discharge summar*" OR "clinical vignette*") |

**Table S2. Risk of Bias**

| **Study** | **Participants** | **Predictors** | **Outcome** | **Analysis** | **Overall Risk of Bias** | **Applicability Concerns** | **Reasoning** |
| --- | --- | --- | --- | --- | --- | --- | --- |
| Liu et al., 2024 (China) [24] | Unclear | Low | Unclear | High | High | Unclear | Participant selection and outcome verification incompletely reported, no calibration assessment, missing-data handling not reported despite external validation. |
| Engelstein et al., 2025  (Israel) [25] | High | Low | Low | High | High | High | Fine-tuning on only 20 ECGs, no independent validation cohort, no external validation, no calibration assessment. |
| Fujita et al., 2025  (Japan) [26] | High | High | High | High | High | High | Keyword-based participant selection, incorporation bias, no independent reference standard, no external validation, no calibration. |
| Guan et al., 2025  (USA) [27] | High | Low | Low | High | High | High | Single-center EHR, keyword-enriched training dataset, no external validation, calibration not assessed. |
| Iinuma et al., 2025  (Japan) [28] | High | Low | Unclear | High | High | High | Single-center image dataset, unclear diagnostic reference standard, no external validation, no calibration assessment. |
| Mansoor et al., 2025  (USA) [29] | High | Low | High | High | High | High | Small development dataset, outcome based on clinician differential diagnoses rather than confirmed diagnoses, no external validation, no calibration. |
| Sha et al., 2025 (Macao, China) [30] | High | Low | Low | High | High | Low | UK Biobank selection bias, exclusion of psychiatric comorbidity, no external validation, no calibration assessment. |
| Lammert et al., 2024  (Germany) [31] | Low | Low | Low | High | High | Low | Evaluation used fictional oncology cases with expert comparison but lacked external validation and prospective assessment in clinical workflows. |
| Rau et al., 2024 (Germany) [32] | Low | Low | Low | High | High | Low | Proof-of-concept evaluation using a limited synthetic case set with no external validation and no assessment in routine clinical populations. |
| Zelin et al., 2024 (USA) [33] | Low | Low | Low | High | High | Low | Diagnostic performance was evaluated on a small number of published case reports without external validation or clinical implementation testing. |
| Alexandrou et al., 2025  (USA) [34] | Low | Low | Low | High | High | Low | Performance was evaluated using guideline-based examination questions rather than real clinical populations, with no external clinical validation. |
| Aminan et al., 2025  (USA) [35] | Unclear | Unclear | Unclear | Unclear | Unclear | Unclear | Perspective article; no original prediction model development or validation study was reported. |
| Chen et al., 2025  (China) [36] | Low | Low | Low | High | High | Low | The study used a small, internally generated evaluation dataset without external validation or assessment in real-world clinical settings. Performance was evaluated only on expert ratings, increasing risk of optimistic estimates. |
| Cremaschi et al., 2025  (Italy) [37] | Low | Low | Low | High | High | Low | Validation relied primarily on DSM-5 clinical cases and expert review without external prospective evaluation or assessment of clinical implementation. |
| Fink et al., 2025 (Germany) [38] | Low | Low | Low | High | High | Low | Proof-of-concept study based on a small number of generated radiology reports with no external validation or prospective clinical testing. |
| Gaber et al., 2025  (Germany) [39] | Low | Low | Low | High | High | Low | Evaluation relied on curated retrospective cases without prospective or external validation, and model calibration or clinical impact analyses were not reported. |
| Noll et al., 2025 (Germany) [40] | Low | Low | Low | High | High | Low | Evaluation was limited to 100 clinical cases and physician ratings without external validation or demonstration of clinical performance across diverse settings. |
| Ong et al., 2025 (Singapore) [41] | Low | Low | Low | High | High | Low | Evaluation was conducted using standardized clinical vignettes and pharmacist assessment without prospective external validation in clinical practice. |
| Ozmen et al., 2025  (USA) [42] | Low | Low | Low | High | High | Low | Performance was assessed using standardized clinical queries only, with no independent external validation or real-world clinical evaluation. |
| Ozmen et al., 2025  (USA) [43] | Low | Low | Low | High | High | Low | Performance was assessed using standardized clinical queries only, with no independent external validation or real-world clinical evaluation. |
| Thaker et al., 2025 (USA) [44] | Low | Low | Low | High | High | Low | Validation relied on examination questions rather than clinical patient cohorts, with no external prospective validation of clinical performance. |
| Tung et al., 2025 (Singapore) [45] | Low | Low | Low | High | High | Low | Assessment was performed using fictional outpatient scenarios with comparison to junior clinicians but without prospective external validation. |
| Rewthamrongsris et al., 2026 (Thailand) [46] | Low | Low | Low | High | High | Low | Evaluation used a predefined question set and a small pilot study without external validation or demonstration of performance in routine clinical practice. |
| Li et al., 2026  (USA) [47] | Low | Low | Low | High | High | Low | Performance was evaluated on benchmark datasets without external clinical validation or assessment of calibration or clinical implementation. Comparisons focused on accuracy rather than prediction model performance in practice. |
| Gao et al., 2025 (China) [48] | Low | Low | Low | Unclear | Unclear | Low | Human evaluation and comparative testing are reported, but key prediction-model analytical details are insufficiently described. |
| Gao et al., 2025 (China) [49] | Low | Low | Low | Unclear | Unclear | Low | Human evaluation and comparative testing are reported, but key prediction-model analytical details are insufficiently described. |
| Kwon et al., 2025  (South Korea, USA) [50] | Low | Low | Low | Unclear | Unclear | Low | Analysis methods for prediction model evaluation are incompletely reported, with insufficient information on handling of optimism, calibration, and other key PROBAST-AI analysis elements. |
| Lahiri et al., 2025  (Canada) [51] | High | High | High | High | High | High | This is a retrieval-augmented question-answering/clinical information synthesis system rather than a clinical prediction model, so PROBAST-AI domains are not appropriately addressed. |
| Lin et al., 2025 (USA, China) [52] | Low | Low | Low | Unclear | Unclear | Low | Key analytical details required for PROBAST-AI assessment are insufficiently reported despite multicohort evaluation. |
| Song et al., 2025  (China) [53] | Low | Low | Low | Low | Low | Low | Internal and external validation are reported with appropriate evaluation cohorts and no major concerns explicitly identified from the reported methodology. |
| Wu et al., 2025 (China, USA) [54] | Low | Low | Low | Unclear | Unclear | Low | Retrospective evaluation is reported with performance metrics, but important analytical details remain insufficiently reported. |
| Wu et al., 2025 (Taiwan) [55] | Low | Low | Low | Unclear | Unclear | Low | Retrospective evaluation is reported with performance metrics, but important analytical details remain insufficiently reported. |
| Cypko et al., 2026  (Germany) [56] | High | High | High | High | High | High | This is primarily a decision-support/usability study rather than development or validation of a multivariable prediction model, making several PROBAST-AI domains high risk or not appropriately addressed. |
| Hashjin et al., 2026 (Iran, Australia) [57] | Low | Low | Low | Unclear | Unclear | Low | External evaluation is reported, but insufficient methodological detail is provided regarding prediction model analysis, calibration, and handling of model performance assessment. |
| He et al., 2026 (China) [58] | Low | Low | Low | Unclear | Unclear | Low | The study reports model development and evaluation but lacks sufficient reporting of analytical methods required for a low-risk PROBAST-AI judgment. |

**Table S3. Characteristics of included studies evaluating post-training and retrieval strategies to improve clinical large language model performance**

| **Study year (country)** | **Study design and clinical context** | **Base model(s), openness, scale, and modality** | **Primary LLM Improvement strategy** | **Post-training/retrieval details** | **Prompting strategy** | **Clinical performance and key results** | **Brief conclusion** |
| --- | --- | --- | --- | --- | --- | --- | --- |
| **Fine-tuning / SFT / PEFT** | | | | | | | |
| Liu et al., 2024 (China) [24] | In-silico benchmark, model development using cfDNA sequencing datasets; Oncology, liquid biopsy, cancer detection. | LLaMA-2-7b-hf (instruction-tuned as iLLMAC); open-source; 7B; text-only | Instruction tuning/SFT | CfDNA end-motif, fragmentation-profile, and motif diversity score representations from WGS, bisulfite (2451 individuals across public and external datasets). Instruction tuning of LLaMA with demonstration data (2451 individuals total; exact training split not fully). | Instruction template to classify input sequence as cancer or control. | Cancer diagnosis/HCC detection from cfDNA: Internal AUROC 0.886 for cancer diagnosis and 0.956 for HCC detection with 64 end-motifs; external AUROC 0.912 for cancer and 0.938 for HCC. External accuracy 0.865 for cancer diagnosis and 0.905 for HCC detection. | Instruction-tuned LLaMA can accurately classify cfDNA fragmentome-derived text representations and outperformed benchmark methods across several sequencing modalities. |
| Engelstein et al., 2025  (Israel) [25] | Retrospective single-center diagnostic comparison study; Cardiology, ECG-based detection of reduced left ventricular ejection fraction. | GPT-4o Fine-Tuned Vision; proprietary; multimodal | SFT/vision fine-tuning | Labeled 12-lead ECG image examples from the same tertiary-center population (20 training ECG images in primary experiment (10 positive, 10 negative)). OpenAI fine-tuning of GPT-4o Vision using JSONL image-text-label examples (20 labeled ECGs for fine-tuning; evaluation cohort 202). | Structured prompt asking the model to classify whether LVEF was <=35% or >35% and explain briefly. temperature 1; 4-run inference. | LVEF <=35% from ECG images: GPT-4o-FTV accuracy 79.9%, sensitivity 72.9%, specificity 80.8%, PPV 34.0%, F1-score 46.4%; CNN comparator accuracy 89.1%. Clinicians: accuracy 74.9%, sensitivity 65.6%, specificity 76.1%, PPV 27.9%, F1-score 39.0%. | GPT-4o Fine-Tuned Vision showed clinically meaningful ECG-based screening performance and outperformed average clinician assessment, although the dedicated CNN remained superior. |
| Fujita et al., 2025  (Japan) [26] | Retrospective report-classification study with temporal external test; Radiology report surveillance, stroke, neuroradiology, emergency radiology. | Japanese BERT v3 (Tohoku University bert-base-japanese); open-source; ~110M; text-only | SFT | Head CT and MRI free-text radiology reports containing the keyword cerebral infarction (5,573 training reports; 1,883 validation; 834 test). AutoModelForSequenceClassification fine-tuning for 3-class report classification (5,573 training reports before undersampling). | NA (report classification model rather than prompted generative system). | Newly identified acute to subacute brain infarction: Test sensitivities: group 0 0.891, group 1 0.905, group 2 0.959; macrosensitivity 0.918; accuracy 0.923; AUROC for newly identified infarcts 0.979. Average prediction time 0.115 ± 0.037 s per patient. | A fine-tuned Japanese BERT model can accurately and rapidly flag newly identified acute to subacute brain infarcts from free-text CT/MRI reports. |
| Guan et al., 2025  (USA) [27] | Retrospective model development and in-silico evaluation using EHR notes; EHR-based screening across outpatient and inpatient notes; cognitive decline, ADRD, geriatrics, general internal medicine. | GatorTron-based CD-Tron; not clearly reported; Billions; exact parameter count not reported in the extracted snippets; text-only | SFT | Mass General Brigham deidentified clinical notes/note sections (2,166 patients over 4 years; Dataset I = 4,949 labeled sections). Task-specific fine-tuning with classification head on top of large clinical language model (4,949 labeled sections). | No prompt-based inference for CD-Tron; GPT-4 comparator used structured prompts. | Cognitive decline detection from clinical notes: Precision 0.8812; Recall 0.9826; F1 0.9289; ROC-AUC 0.9993; PR-AUC 0.9804. GPT-4 baseline: Precision 71.7%, Recall 91.4%, F1 80.3%. | A fine-tuned large clinical language model substantially improved early cognitive decline detection from EHR notes over traditional NLP baselines and provided clinically useful interpretability via SHAP. |
| Iinuma et al., 2025  (Japan) [28] | In-silico benchmark, retrospective image dataset model development, validation, and independent held-out test; Dermatology, pigmented skin lesion image classification from routine clinical photographs. | InstructBLIP-flan-t5-xl; open-source/open-weight; Not explicitly reported; FLAN-T5-XL language backbone (~3B) implied; multimodal (clinical photographs + text prompts) | SFT/multimodal fine-tuning | Curated image-text dataset of manually cropped lesion ROIs from clinical photographs across 8 pigmented skin lesions (979 usable samples; train 80%/validation 20%). Seq2Seq fine-tuning with Hugging Face Seq2SeqTrainer; label masking; image resize to 224×224 (979 image-text pairs). | Instruction-style visual question-answer format (“USER … ASSISTANT”). | Eight-class lesion classification from standard clinical: Independent test: macro precision 86.1%, recall/sensitivity 86.0%, specificity 98.2%, F1 0.860; macro ROC AUC 0.921. Validation: precision 90.6%, recall 90.3%, specificity 98.5%, F1 0.903. | A fine-tuned multimodal LLM can classify common pigmented skin lesions accurately from routine clinical photographs alone, suggesting utility where dermoscopy is unavailable. |
| Mansoor et al., 2025  (USA) [29] | Multicenter retrospective cohort study; Rural pediatric primary care, pediatrics, differential diagnosis support. | GPT-3 (DaVinci version); proprietary; text-only | SFT | Deidentified rural pediatric EHR encounters with pediatrician-generated differential diagnoses (500 total encounters; 350 train/150 test). Fine-tuning via OpenAI API on concatenated clinical text to generate up to five differentials (350 training encounters). | Concatenated clinical text input; model asked to generate up to five differential diagnoses. | Differential diagnosis accuracy: Accuracy 87.3% (131/150); sensitivity 85%; specificity 90%; comparable to pediatricians 91.3% (137/150), P=.47. Age-group accuracy: 0-5y 87%, 6-12y 89%, 13-18y 86%. | Fine-tuned GPT-3 provided pediatric differential diagnosis support comparable to pediatricians for common presentations in rural settings, though broader validation is needed. |
| Sha et al., 2025 (Macao, China) [30] | In-silico benchmark, retrospective cohort-based model development and evaluation; Population biobank, mental health, psychiatry (major depressive disorder diagnosis). | LLaMA 3.1 8B; LLaMA 3.1 70B; open-source; text-only | SFT + PEFT (LoRA/QLoRA) | UK Biobank tabular cohort data converted into instruction prompts (274,348 participants (12,715 MDD; 261,633 controls)). Instruction-style supervised fine-tuning with LoRA (main) and QLoRA comparison (274,348 total records; exact train/validation/test split not). | Instruction prompt with tabular-to-text conversion; List Template, Text Template, GPT Generation Template tested. | Major depressive disorder: Best model (MDD-LLM 70B): Accuracy 0.8378; F1 0.8184; AUC 0.8919 (95% CI 0.8799–0.9040). Specificity 0.8358. | Large-scale supervised fine-tuning of LLaMA on UK Biobank tabular-to-text data outperformed conventional ML/DL baselines for MDD diagnosis; LoRA was slightly better than QLoRA and the model remained relatively robust to missing data. |
| **Retrieval-Augmented Methods** | | | | | | | |
| Lammert et al., 2024  (Germany) [31] | In-silico benchmark with iterative expert feedback from a molecular tumor board; Precision oncology, molecular tumor board decision support. | Gemini Pro (Google); proprietary; text-only | RAG + CoT + expert-guided domain knowledge | PubMed full-text articles, PubMed-indexed clinical studies on drug response in tumor-specific contexts (10 fictional oncology case vignettes; corpus size not explicitly reported). No supervised fine-tuning reported; system-level iterative enhancement after MTB feedback. Retrieval used PubMed via PyMed; clinical studies; ClinicalTrials.gov and; Vertex AI Search vector store/cloud storage retriever. | Chain-of-thought with four prompts to summarize literature, identify guidelines, identify trials, and synthesize recommendations. | Personalized treatment recommendations with MTB experts: Enhanced system concordance 94.7%; semantic cosine similarity improved from 0.71 to 0.76 (P=.01). Median proposed treatment options 4 for enhanced system vs 2 for MTB experts. | Expert feedback and domain-specific retrieval substantially improved LLM performance for precision oncology decision support, yielding high concordance with MTB recommendations. |
| Rau et al., 2024 (Germany) [32] | Prospective proof-of-concept in-silico evaluation using synthetic case files; Gastrointestinal radiology, differential diagnosis from imaging descriptions. | GPT-4 (06-13 version) as generic chatbot and gastrointestinal imaging-aware chatbot (GIA-CB); proprietary; text-only | RAG/zero-shot retrieval-augmented prompting | RadioGraphics Top 10 Reading List for gastrointestinal imaging (96 peer-reviewed documents). Retrieval used Curated peer-reviewed gastrointestinal imaging literature; LlamaIndex v0.8.4; OpenAI text-embedding-ada-002-v2. | Zero-shot precision prompt requesting main and two additional differential diagnoses and an explanation. temperature 0; top 15 retrieved. | Main differential diagnosis: Main diagnosis matched in 39/50 cases (78%) for GIA-CB vs 27/50 (54%) for generic GPT-4. Main diagnosis contained within top-3 differentials 90% vs 74%. | A gastrointestinal imaging-aware GPT-4 chatbot using retrieval augmentation improved differential diagnosis accuracy and provided traceable source excerpts, supporting more trustworthy radiology decision support. |
| Zelin et al., 2024 (USA) [33] | In-silico benchmark; Rare disease diagnostic decision support, genetics, general medicine. | ChatGPT 3.5 (RareDxGPT augments GPT-3.5); proprietary; text-only | RAG | RareDis Corpus (717 rare diseases in RareDis Corpus). Retrieval used RareDis Corpus text documents converted to markdown; OpenAI embeddings; LangChain cosine similarity; FAISS vector database. | Three prompt types tested: Prompt; Prompt and Explanation; Prompt and Role Play. | Diagnosis generation for rare diseases: Best RareDxGPT accuracy 43% with Prompt + Explanation; 40% with Prompt; 40% with Prompt + Role Play. Global Quality Scale (GQS): RareDxGPT averages 2.67 (Prompt), 2.59 (Prompt + Explanation). | Adding a rare-disease knowledge base through RAG modestly improved GPT-3.5 rare disease diagnosis, and prompting strategy materially affected performance. |
| Alexandrou et al., 2025  (USA) [34] | In-silico benchmark; Cardiology, interventional cardiology, acute coronary syndrome guideline-based decision support. | ChatGPT-4o; DeepSeek R1; Med-PaLM 2; mixed; text-only | RAG | 2020 ESC acute coronary syndrome guideline companion document (ESC guideline PDF corpus; evaluation used 38 open-ended questions). Retrieval used 2020 ESC ACS guidelines; Chunk size 500 tokens; sentence-transformer all-MiniLM-L6-v2 embeddings; FAISS. | Zero-shot; same prompt structure across models. Temperature 1. | Guideline-based ACS question answering: DeepSeek R1 + RAG 94.7%; ChatGPT-4o + RAG 92.1%; DeepSeek R1 no RAG 78.9%; ChatGPT-4o no RAG 71.1%; Med-PaLM 2 68.4%. Median similarity scores. | Embedding current guideline content through RAG substantially improved LLM alignment with ACS recommendations, especially for ChatGPT-4o and DeepSeek R1. |
| Aminan et al., 2025  (USA) [35] | In-silico benchmark; Ophthalmology, glaucoma assessment and question answering. | GPT-4.5-PREVIEW integrated with R2R; mixed; text-only | RAG | Glaucoma-specific textual knowledge base (1,859 peer-reviewed articles + 15 guidelines + 3 glaucoma textbooks). Retrieval used Glaucoma textbooks, guidelines, and peer-reviewed articles; R2R; OpenAI text-embedding-3-large; Postgres-backed HNSW cosine index. | Single-turn prompts for case reports and multiple-choice items. | Diagnostic accuracy/correct glaucoma question answering: 81.8% accuracy on case reports; 91.2% accuracy (228/250) on BCSC items. RAGAS: faithfulness 0.70. | A glaucoma-specialized RAG pipeline improved evidence-grounded ophthalmic question answering over non-RAG baselines and showed promise as expert-level decision support. |
| Chen et al., 2025  (China) [36] | In-silico comparative evaluation of a RAG-based clinical decision framework using expert-scored patient complaint cases; Rehabilitation medicine, chronic low back pain treatment decision support. | GPT-4.0 (core CLBP-GPT system); comparators GPT-4.0 baseline, ERNIE Bot, DeepSeek; proprietary; text-only | RAG + prompt engineering (least-to-most/LtM); no supervised fine-tuning reported | Knowledge base built from latest CLBP-related PubMed papers and medical guidelines (4,612 CLBP-related articles/guidelines (2021–2025)). None reported; system-level retrieval augmentation and prompt engineering only. Retrieval used PubMed research papers and clinical guidelines on chronic low back pain; Professional vector knowledge base with cosine-similarity retrieval. | Least-to-most (LtM)/progressive prompt engineering to simulate expert decision-making. | Expert-scored quality of personalized CLBP treatment: Overall mean score 4.40 (SD 0.20), higher than GPT-4.0 4.03 (SD 0.48), ERNIE Bot 3.54 (SD 0.53), and DeepSeek 3.81 (SD 0.47). Accuracy dimension 4.38 (SD 0.19). | Integrating GPT-4.0 with CLBP-specific RAG and LtM prompting improved the quality of personalized treatment recommendations compared with general LLM baselines. |
| Cremaschi et al., 2025  (Italy) [37] | In-silico benchmark with manual expert validation; Mental health decision support, psychology, psychiatry. | Gemma 2; open-source; 27B; text-only | RAG | ICD-11 Chapter 6 and ICD-11-CDDR diagnostic content merged into disorder prompts (719 disorder records; 55 with at least one inclusion). Retrieval used ICD-11 and ICD-11-CDDR; LangChain embeddings; ChromaDB. | Clinician/patient case description entered into chat; retrieved disorder prompt context supplied to model. | Diagnosis/differential diagnosis support in mental health: Accuracy 76.0% (reported as 76%/76.1%). Manual expert validation. | An ICD-11-grounded Gemma-2 RAG system can support mental health diagnostic reasoning with moderate accuracy, but clinician oversight remains essential given the residual error rate. |
| Fink et al., 2025 (Germany) [38] | Prospective proof-of-concept study using synthetic radiology-report findings; Emergency radiology, trauma radiology, diagnosis and injury classification. | GPT-4 Turbo (TraumaCB augments GPT-4 Turbo); proprietary; text-only | RAG | RadioGraphics top ten reading list for trauma radiology (70 peer-reviewed articles; evaluation used 100 report findings derived from). Retrieval used RadioGraphics top ten trauma radiology reading list; OpenAI text-embedding-ada-002-v2; sentence windows (+/-5 sentences). | Two-step prompt: primary diagnosis first, then classification/grading using diagnosis-specific retrieved context. Temperature 0. | Diagnosis, injury-classification, and grading accuracy: TraumaCB: 100% correct diagnoses, 96% correct classification, 87% correct grading; generic GPT-4: 93%, 70%, and 48%, respectively. Median explanation rating 5.0 and median trust rating 5.0 (IQR 0.0). | Adding curated trauma-radiology knowledge to GPT-4 substantially improved diagnostic, classification, and grading performance and increased user trust. |
| Gaber et al., 2025  (Germany) [39] | Benchmark evaluation of LLM workflows on curated real-world cases; Clinical decision support for triage, specialty referral, and diagnosis using ED, ICU-derived cases. | Claude 3.5 Sonnet; Claude 3 Sonnet; Claude 3 Haiku; RAG-assisted Claude 3.5 Sonnet; proprietary; text-only | RAG/agentic workflow | 30 million PubMed abstracts converted to embedding vectors (30 million PubMed abstracts; evaluation dataset 2,000 MIMIC-IV-derived). Retrieval used PubMed abstract corpus; Semantic vector retrieval over 30M PubMed abstracts in a high-dimensional vector. | Task-specific prompts for triage, specialty referral, and diagnosis under general-user and clinician-user scenarios. | Accuracy for triage, specialty referral, and diagnosis: RAG-assisted model (clinical-user setting): triage exact-match 65.75%, triage-range 77.15%, specialty matched 77.28%, specialty top-3-at-least-one 86.45%. Claude 3.5 Sonnet without RAG achieved the highest overall average accuracy. | Across 2,000 curated MIMIC cases, LLM workflows could support triage, referral, and diagnosis, but RAG assistance did not uniformly outperform the best standalone Claude workflow. |
| Noll et al., 2025 (Germany) [40] | Technical proof-of-concept, in-silico benchmark with physician review; Emergency department, internal medicine, cardiology and gastroenterology decision support. | Mixtral-8x7B Instruct; open-weight; 8x7B MoE; text-only | RAG | German cardiology and gastroenterology guidelines and specialist articles, textbook chapters (Knowledge compendium size NR; evaluation on 100 German clinical cases). Retrieval used German guideline/text compendium for cardiology and gastroenterology; Sparse BM25 vs semantic retrieval; semantic workflow used embedding vectors in a. | German emergency-department precision prompt with or without retrieved context. Temperature 0. | Guideline retrieval relevance and emergency-care decision: Semantic retrieval reduced irrelevant guideline selection and was faster than sparse retrieval (<3 s per case). Formal errors in 23% of semantic-retrieval responses, 14% of sparse-retrieval responses. | Semantic retrieval improved guideline selection and latency, but automated chunking errors limited downstream answer quality, so the non-retrieval Mixtral model remained best in physician-rated usefulness. |
| Ong et al., 2025 (Singapore) [41] | Prospective, crossover, open-label study; Acute care, inpatient medication review across 16 medical and surgical specialties; pharmacy, medication safety. | Gemini Flash; Gemini 1.5 Pro; GPT-4 Turbo; GPT-4o; Claude 3.5 Sonnet (selected for co-pilot arm); proprietary; text-only | RAG (no parameter fine-tuning) | Indexed pharmacological knowledge including local institutional monographs, dosing protocols, and drug-use guidelines (91 prescribing error scenarios within 40 complex vignettes across 16). Retrieval used Drug monographs, dosing protocols, local institutional knowledge; LlamaIndex with auto-merging retrieval. | Chain-of-thought prompts standardized to SOAP format; pharmacist responses in SBAR format. triplicate runs. | Drug-related problems/prescribing errors: Co-pilot mode accuracy 61% (precision 0.57, recall 0.61, F1 0.59) vs pharmacist alone 46% and LLM alone 51%. Co-pilot improved detection of errors causing serious harm by 1.5-fold over pharmacist alone. | LLM-based CDSS can augment pharmacists in co-pilot mode for medication safety review, but RAG did not consistently improve all models and further refinement is needed. |
| Ozmen et al., 2025  (USA) [42] | System development and computational proof-of-concept evaluation; Hand surgery clinical decision support. | OpenAI o3-mini; OpenAI text-embedding-ada-002; mixed (proprietary llm/embeddings with open-source retrieval stack); Not disclosed; text-only | RAG only | Open-access peer-reviewed hand surgery literature from PubMed Central (4,510 articles (2000–2024); evaluation with 15 standardized clinical queries). None reported. Retrieval used PubMed Central open-access hand surgery publications; RAPTOR hierarchical clustering with UMAP and GMM. | Query expansion (up to 5 subqueries), top-k=3 semantic retrieval, literature-grounded response synthesis with citations. | Evidence-based response correctness: Average G-Eval correctness 0.79. Average SEM similarity 0.75 (range 0.54–0.86) and maximum similarity 0.80 (range 0.56–0.91). | HandRAG provided literature-grounded, clinically meaningful responses across standardized hand surgery queries, supporting feasibility as a point-of-care evidence support tool. |
| Ozmen et al., 2025  (USA) [43] | Tool development and in-silico benchmark using standardized clinical scenarios; Microsurgery, plastic surgery, reconstructive surgery clinical decision support. | OpenAI o1-mini; proprietary; text-only | RAG | Open-access peer-reviewed microsurgical literature from PubMed Central (4,876 publications (2000-2024)). Retrieval used PubMed Central microsurgical publications; OpenAI embeddings; RAPTOR-style hierarchical clustering. | Natural-language clinical query with RAG context and citation-linked response generation. | Microsurgery clinical query answering: Answer relevancy 0.953 (range 0.857-1.000); faithfulness 0.907 (range 0.676-1.000); G-Eval correctness 0.88. SEM similarity score 0.75. | A microsurgery-specific RAG tool built on the open-access literature produced highly relevant, literature-grounded clinical recommendations and may support point-of-care decision-making and education. |
| Thaker et al., 2025 (USA) [44] | In-silico benchmark study of zero-shot versus RAG-augmented LLM workflows; Radiation oncology, knowledge-intensive clinical decision support and training benchmark. | RadOncRAG (Iridium Model) applied to leading foundation LLMs including GPT-4o, Claude-3.5-Sonnet, Llama-3-70b, Llama-3.1-405b, Gemini-2.0; reasoning models tested separately; hybrid (open and proprietary models benchmarked); Varied across models (7B to 405B; reasoning models also evaluated); text-only | RAG | Radiation oncology textbooks, clinical guidelines, protocols, Q&A archives, and research materials. Retrieval used Specialized radiation oncology database including Gunderson and Tepper; OpenAI text-embedding-3-small (1536 dimensions); plain-text sliding window 500 tokens. | Zero-shot baseline compared with augmented prompts containing retrieved top 5 context chunks; majority-voting analysis also performed. top 5 retrieved. | Benchmark accuracy on ACR in-training examination: RAG improved Gemini-2.0 from 72.5% to 79.2%, Llama-3-70b from 67.4% to 75.8%, and GPT-4o from 79.9% to 85.6%. Domain-specific performance in clinical, statistics, biology, and physics domains. | A radiation-oncology-specific RAG pipeline improves nonreasoning LLM benchmark performance by grounding answers in domain evidence, while reasoning models already perform strongly and do not gain from RAG. |
| Tung et al., 2025 (Singapore) [45] | In-silico comparative benchmark using fictional outpatient scenarios and junior clinician comparison; Urology, outpatient prostate cancer screening and PSA testing decision support. | RAG-enabled LLM pipeline using OpenAI and Anthropic API components (specific final frontier model not explicitly disclosed); proprietary apis; Not disclosed; text-only | Guideline-grounded RAG (no fine-tuning) | EAU and AUA prostate cancer early-detection guidelines, and LLM-based Charlson Comorbidity Index life-expectancy (44 fictional outpatient case scenarios; guideline documents 239 pages). None reported. Retrieval used European Association of Urology and American Urological Association; Unstructured API for PDF ingestion; OpenAI embeddings; Qdrant vector DB. | Advanced prompting with CoT reasoning, context filtering, expert clinician persona, and example output structures. | Guideline-concordant PSA testing recommendation accuracy: RAG-LLM accuracy 95.5% concordance (210/220) vs junior clinicians 62.3% closed-book and 74.1% open-book. Fleiss κ 0.294 closed-book and 0.321 open-book. | A guideline-grounded RAG-LLM substantially outperformed junior clinicians in simulated PSA testing decisions and may improve consistency and efficiency of urologic screening recommendations. |
| Rewthamrongsris et al., 2026 (Thailand) [46] | In-silico comparative benchmark with pilot educational user study; Dentistry, oral surgery, infective endocarditis prophylaxis for dental procedures. | GPT-4.5 Preview; GPT-o3-mini; Claude 3.7 Sonnet; Gemini 2.0 Pro; DeepSeek Reasoner; Llama 3 70B; Grok 3 beta; plus legacy GPT-4o, Claude 3.5 Sonnet, Gemini 1.5 Pro; mixed (proprietary and open-weight); Mixed/mostly undisclosed; includes Llama 3 70B; text-only | RAG only (no SFT) | 2021 AHA infective endocarditis prophylaxis guideline converted into 117 semantically coherent chunks (117 guideline chunks; 28 true/false questions). None reported. Retrieval used 2021 American Heart Association infective endocarditis guideline; MiniLM-L6-v2 embeddings and FAISS similarity search. | With and without preprompt (“You are an experienced dentist.”). | Guideline-based infective endocarditis prophylaxis question: Best accuracy 90.0% with Grok 3 beta + preprompt; best no-preprompt accuracy 83.6% with DeepSeek Reasoner. Consistency up to 96.4%. | RAG and prompt engineering improved LLM performance on IE prophylaxis questions, but current real-world educational utility remains limited. |
| Li et al., 2026  (USA) [47] | In-silico benchmark and performance evaluation; Sleep medicine, diagnostic reasoning (MCQs and clinical vignettes). | Llama-3 (8B, 70B), Qwen 2.5 (14B, 235B); open-source; 8B–235B; text-only. | RAG (comparing corpus structure, retrieval strategy, and pipeline complexity). | Five sleep medicine textbooks. GPT-4o-assisted cleaning (boundary repair). TOC-aligned structured segments (~512 tokens) vs. raw text. Hybrid indexing (BM25 + PubMedBERT-dense) with RRF. | textbooks. GPT-4o-assisted cleaning (boundary repair). TOC-aligned structured segments (~512 tokens) vs. raw text. Hybrid indexing (BM25 + PubMedBERT-dense) with RRF.  Baseline (direct concatenated context) vs. Augmented (query rewriting [≤30 words] and post-retrieval passage screening). | MCQ Accuracy: Improved across all models; Llama 8B (+10.6%), Qwen 235B (peak 87.3%). Clinical Cases: Llama 8B accuracy dropped 7.1% (Raw-Dense) due to noise. Structured hybrid reversed this, yielding a 6.1% mean Top-1 accuracy gain. | RAG effectiveness is scale-dependent; structured corpora and hybrid retrieval are essential to prevent distraction in smaller models and ensure clinical stability. |
| **Hybrid / Multicomponent Methods** | | | | | | | |
| Gao et al., 2025 (China) [48] | Retrospective multimodal model development and benchmark evaluation with expert comparison; Oral and maxillofacial surgery, dentistry, panoramic radiography-based osteonecrosis of the jaw support. | Qwen2-VL (core MLLM); SwinTransformer; U-Net; open-weight/open-source components; Not explicitly reported; multimodal (panoramic radiography images + clinical records/text queries) | Multimodal SFT (LoRA) + transfer learning + segmentation | Panoramic radiographs and medical records of pathology-confirmed ONJ patients (366 ONJ patients; training 282/validation 10/test 74). LoRA fine-tuning of Qwen2-VL on modified ONJ Q&A data (366 total cases). | Visual Q&A using segmented PR image and query text prompt. | ONJ diagnosis/treatment support responses: Human evaluation accuracy 0.9598 (95% CI 0.9351–0.9845), outperforming junior OMFSs 0.8835 (95% CI 0.8432–0.9238) and approaching senior OMFSs 0.9959. Text generation metrics: BLEU-4 0.5260, METEOR 0.8436, ROUGE-1 0.7345. All on scale of 0-1. | The ONJ-TS multimodal system integrated imaging and text to provide accurate ONJ diagnosis and treatment support, outperforming junior surgeons and supporting clinical decision-making. |
| Gao et al., 2025 (China) [49] | Dual-center retrospective model-development and external-validation study; Perioperative, postoperative complication detection and severity grading, anesthesiology and surgical perioperative care. | Open-source Qwen 3 models (4B, 8B, 14B, 32B; PeriComp variants after optimization/LoRA), benchmarked with commercial and other open-source models; primarily open-source for optimized deployable models; commercial comparators also evaluated; 4B, 8B, 14B, and 32B for main optimized models; text-only | Prompt optimization + CoT + task decomposition + LoRA | Dual-center perioperative clinical narratives, postoperative notes, abnormal laboratory results (Center 1 total n=146 cases (100 used for fine-tuning). LoRA/SFT with targeted single-complication prompts (100 training cases from Center 1; targeted strategy yields). | Structured JSON output; CoT prompting with "think" field; targeted single-complication assessment prompts; comprehensive. temperature 0; 5 repeated runs. | Micro-F1 for perioperative complication detection and severity: On external validation, optimized 4B model micro-F1 improved from 0.28 to 0.64; optimized 8B model exceeded 0.70 and surpassed human experts (F1=0.69). Precision and recall. | Targeted task decomposition and LoRA can convert small locally deployable open-source LLMs into expert-level tools for perioperative complication detection while preserving privacy and data sovereignty. |
| Kwon et al., 2025  (South Korea, USA) [50] | Domain-specific model development and benchmark evaluation on a pre-collected dataset; Ophthalmology, diagnosis and treatment planning. | Ophtimus-V2-Tx/Ophtimus-V2-Base (8B ophthalmology-specialized foundation model); open-weight; text-only | Continued domain pretraining + instruction tuning + case-report SFT/PEFT | Domain-specific textbooks and PubMed ophthalmology articles for base pretraining (Base pretraining corpus included textbooks (4M) and PubMed ophthalmology). LoRA-based PEFT on 8B model with additional case-report fine-tuning for treatment planning (>10,000 case reports). | Schema-structured case inputs preserving laterality, measurements, test names, and findings. temperature 0. | Clinically grounded diagnosis and treatment: ROUGE-L 0.40, BLEU 0.26, METEOR 0.45, SEMSCORE 0.80; best secondary-diagnosis full-code score 0.15; medication full-code 0.31; surgical full-code 0.13. Primary diagnosis CliBench: L2 0.58 and L4 0.40. All on scale of 0-1. | A compact ophthalmology-specific model fine-tuned on structured case reports approached or exceeded a large general model on selected clinically grounded diagnosis/treatment tasks while remaining deployable on-premises. |
| Lahiri et al., 2025  (Canada) [51] | System development, benchmark evaluation, and clinical use-case demonstration; Alzheimer disease, biomedical literature retrieval for clinical use cases. | Llama-2-7b-pubmed; LLaVA v2 with Llama-2 7B backbone; open-source/open-weight; 7B text backbone; 7B multimodal backbone; multimodal (text, tables, images from pubmed articles) | Multimodal RAG + PEFT/QLoRA | Top 2,000 PubMed articles on Alzheimer’s disease including full text, abstracts, tables, and figures (2,000 PubMed articles; benchmarked on BioASQ and PubMedQA). QLoRA fine-tuning of Llama-2-7b-pubmed and fine-tuning of LLaVA v2 (PubMedQA size not stated in article text). Retrieval used PubMed biomedical literature (text, tables; FaissDB and object store with cross-modal embedding fusion. | Context-aware multimodal question answering via web app. | Retrieval/synthesis performance for Alzheimer biomedical QA: Comparative model evaluation: Recall 0.88, Precision@10 0.85, F1 0.86. BioASQ: Precision@10 0.71, Recall 0.80, MAP 0.78, QA accuracy 0.72, F1 0.75. | AlzheimerRAG’s multimodal RAG framework improved retrieval and synthesis of Alzheimer-specific biomedical information and was reported to have low hallucination in clinical use-case scenarios. |
| Lin et al., 2025 (USA, China) [52] | Retrospective multimodal model development with external validation; Neurology, Alzheimer’s disease diagnosis using MRI plus structured clinical data. | ADLIP (DenseNet-based 3D encoder + Bio_ClinicalBERT text encoder); mixed/research model built from open components; multimodal | Hybrid multimodal contrastive learning + multi-teacher knowledge distillation | ADNI paired 3D T1 MRI and structured clinical/biomarker data; external HABS-HD cohort (ADNI: 841 participants, 3396 MRI scans; train/val/test = 2403/332/663 scans). Task-specific fine-tuning of multimodal contrastive framework with InfoNCE and MTKD (3396 paired image-text scans). | Zero-shot class prediction by cosine similarity to candidate text prompts; clinical text standardized to <=77 tokens. | Three-class AD/MCI/CN diagnosis and zero-shot prediction: Model reported to outperform baseline CLIP and fine-tuned CLIP in accuracy, F1, and macro-AUC. Longitudinal prediction scores aligned with disease trajectories. | A multimodal contrastive MRI-plus-clinical-text foundation model improved AD diagnostic representation learning, including zero-shot transfer and external generalizability across racial groups. |
| Song et al., 2025  (China) [53] | Retrospective cohort development with internal validation and external validation; Emergency neurology, acute stroke diagnosis and treatment triage. | ChatGLM-6B; open-weight/open-source; text-only (clinical free text + ncct reports, not raw imaging) | Instruction tuning + PEFT (LoRA) + tool chaining | Emergency clinical notes from EHRs and noncontrast CT radiology reports and other examination records (1,885 patients in development set; 335 patients in external test cohorts). Instruction fine-tuning with LoRA after selecting best EHR entry combinations (1,885 cases total, split 9:1 for training/internal validation). | Explicit multi-question diagnostic prompt; zero-shot and 3-shot dynamic few-shot tested. | Stroke diagnosis (stroke vs nonstroke): Fine-tuned model accuracy 99.0% on internal validation and 95.5%/79.1% on two external cohorts. Ischemia vs hemorrhage accuracy 100% internal and 99.1%/97.1% external. | A ChatGLM-6B model leveraging structured clinical text and NCCT reports can accurately identify stroke, subtype, and key reperfusion decisions, showing promise for stroke care support. |
| Wu et al., 2025 (China, USA) [54] | Method-development study combining segmentation benchmarks with pilot clinician-scored evaluation; Liver cancer diagnosis support, oncology, radiology, and radiotherapy. | ChatGLM-6B large language model combined with dedicated liver tumor and liver vessel segmentation small models; open-source/academic framework; 6B for LLM; small model sizes NR; multimodal pipeline (ct imaging + medical records + retrieved knowledge) | Small-model feature extraction + RAG + CoT | Three knowledge types: patient-specific imaging/medical-record knowledge, filtered reliable domain knowledge (NR; LLM evaluation used 10 patient cases). Retrieval used CSCO Guidelines for Diagnosis and Treatment of Primary Liver Cancer; RAG/LangChain using the LLM encoder to vectorize the knowledge base and user. | Liver-cancer-specific CoT prompt mimicking experienced doctors with three-step reasoning over medical records, image-derived features. | Doctor-scored quality of interpretation of medical: Average doctor score for interpretation quality improved from 5.9 to 7.2 and treatment-plan reasonableness from 4.2 to 6.5 vs control. Segmentation performance: tumor DICE 0.912 on 3DIRCADB and 0.876 on private dataset. | Combining image-specific small models, doctor-style CoT prompting, and retrieval from trusted liver-cancer knowledge sources improves the usefulness, reliability, and interpretability of ChatGLM-6B for liver cancer diagnostic assistance. |
| Wu et al., 2025 (Taiwan) [55] | Retrospective evaluation; Radiology, neuroradiology, brain metastasis MRI reporting. | GPT-4o (with ARAMP adaptive RAG workflow); Gemini 2.0 Pro benchmark; proprietary; multimodal | Adaptive RAG/iterative prompt refinement (not parameter fine-tuning) | Radiomic features from AX_T1+C MRI and composite PNG images and five authoritative medical references (200 total cases in radiomic/GPT dataset (100 cancer, 100 matched controls)). NA (authors clarify 'training' was structured prompting/post-training refinement. Retrieval used Five authoritative medical references and guideline-based; Adaptive RAG with prompt-linked indexing; exact embedding/vector database not reported. | Standardized multi-step diagnostic workflow prompt (image definition -> differential diagnosis -> treatment strategy -> follow-up). temperature not. | Brain metastasis detection/reporting on MRI: Sensitivity improved from 0.84 (pre-training) to 0.98 (post-training); mean post-trained inference similarity 67.45%. Inter-inference agreement 30.20%. | An adaptive RAG workflow combining GPT-4o with radiomic features and MRI composites improved sensitivity and reporting quality for brain metastasis interpretation. |
| Cypko et al., 2026  (Germany) [56] | Method-development study with clinician user study; Clinical decision support knowledge engineering, Bayesian network modelling, laryngeal cancer oncology use case. | GPT-4; Mixtral 8x7B; fine-tuned GTE-Large embedding model; hybrid (gpt-4 proprietary; mixtral open-weight); GPT-4 NR; Mixtral 8x7B; GTE-Large NR; text-only | RAG + embedding-model tuning + prompt optimization | Medical guidelines and textbooks on laryngeal cancer; GPT-4-generated synthetic retrieval questions for (Biomedical corpus size NR; 2,070 synthetic questions generated for). No LLM fine-tuning for generation model; fine-tuned GTE-Large embedding model and query (2,070 synthetic questions (30 moderate + 30 difficult). Retrieval used Laryngeal-cancer guidelines and textbooks; Fine-tuned GTE-Large embeddings; recursive/semantic/propositional chunking. | RAGAS-derived prompt templates and Mixtral-based query optimization and BN-modelling suggestion generation. | Retrieval accuracy and BN-modelling usability: Best retrieval accuracy 0.90 with recursive chunking + fine-tuned GTE-Large; semantic chunking improved from 0.75 to 0.85 with query expansion/HyDE. All clinicians created comprehensive BN models within 1 hour. | LLM-RAG support improved retrieval quality and enabled efficient, low-burden expert Bayesian-network modelling, though clinicians still needed to correct occasional causality/idiom violations. |
| Hashjin et al., 2026 (Iran, Australia) [57] | Federated multimodal model development with external cohort evaluation; Dermatology, skin-lesion diagnosis and patient-facing explanation. | PaLI-Gemma 2 vision-language backbone with DeepSeek-V3 as primary explanatory LLM; open-weight/hybrid; ~2.95B active PaLI-Gemma 2 backbone + ~30M trainable LoRA adapters; DeepSeek-V3 explanatory model (MoE, 37B active per inference); multimodal | Multimodal SFT/LoRA + federated learning + trust-aware aggregation + A-RAG | Four private institutional dermatology datasets of biopsy-confirmed clinical and dermoscopic images and curated (28,943 internal training/validation/test images across four nodes (9,930). LoRA fine-tuning of PaLI-Gemma 2 with federated instruction tuning (Internal federated nodes total 28,943 samples). Retrieval used Curated dermatology knowledge base from trusted sources; BAAI bge-small-en-v1.5 sentence-transformer; 384-dimensional embeddings. | Two-stage pipeline: image-based diagnosis and benign/malignant prediction, then patient-style explanatory questions grounded by A-RAG. | Skin-lesion diagnosis and malignancy prediction: Diagnostic accuracy 90.2% across 11 lesion types; malignancy prediction accuracy 93.3%. Per-class sensitivity/specificity/AUC/ECE reported. | A trust-aware federated multimodal dermatology system can deliver strong diagnostic performance with interpretable, retrieval-grounded explanations while preserving privacy. |
| He et al., 2026 (China) [58] | Retrospective EHR-based model development and evaluation study with prototype clinical decision support system; Outpatient diabetes, endocrinology clinical decision support. | Llama 3.1-8B; Qwen3-8B; GLM4-9B; open-source/open-weight; text-only | SFT/PEFT (LoRA) + RAG + agent-assisted data integration | Deidentified outpatient diabetes EHRs including demographics, chief concern, history, diagnosis, medications, tests (20,619 patients in main dataset; additional 300-patient March 2022 clinical). Instruction-based prompting and LoRA fine-tuning on attention layers (20,619 cases; 80% training/20% held-out test). Retrieval used Hospital medical order database, diabetes treatment guidelines. | Instruction templates for tailored clinical recommendations. | Fine-tuned GLM4-9B achieved BLEU-4 67.93 (SD 2.74), ROUGE-1 44.30 (SD 3.91), ROUGE-2 27.34 (SD 1.85), ROUGE-L 37.67 (SD 2.88) (all on a 0–100 scale), with a physician-rated treatment appropriateness score of 4.72. | Fine-tuned compact LLMs, especially GLM4-9B, showed strong promise for personalized outpatient diabetes decision support when combined with hospital knowledge retrieval. |
